# Furandicarboxylic Acid (FDCA): Electrosynthesis and Its Facile Recovery From Polyethylene Furanoate (PEF) via Depolymerization

**DOI:** 10.1002/cssc.202401190

**Published:** 2024-10-28

**Authors:** Gyula Dargó, Dávid Kis, Amália Ráduly, Vajk Farkas, József Kupai

**Affiliations:** ^1^ Department of Organic Chemistry and Technology Budapest University of Technology and Economics Műegyetem rakpart 3. Budapest 1111 Hungary; ^2^ Hungarian Research Network Research Centre for Natural Sciences Institute of Materials and Environmental Chemistry Magyar tudósok körútja 2 Budapest 1117 Hungary

**Keywords:** Biomass valorization, Depolymerization, Electrocatalytic oxidation, 5-hydroxymethylfurfural, Poly(ethylene furanoate)

## Abstract

Replacing fossil fuels with renewable, bio‐based alternatives is inevitable for the modern chemical industry, in line with the 12 principles of green chemistry. 2,5‐Furandicarboxylic acid (FDCA) is a promising platform molecule that can be derived from 5‐hydroxymethyl furfural (HMF) via sustainable electrochemical oxidation. Herein, we demonstrate TEMPO‐mediated electrooxidation of HMF to FDCA in ElectraSyn 2.0 using inexpensive commercially available electrodes: graphite anode and stainless‐steel cathode, thereby avoiding the often cumbersome electrode preparation. Key parameters such as concentration of HMF, KOH, and catalyst loading were optimized by experimental design. Under the optimized conditions, using only a low amount of TEMPO (5 mol %), high yield and Faradaic efficiency of 96 % were achieved within 2.5 h. Moreover, since FDCA is a monomer of the bio‐based poly(ethylene furanoate), PEF, we aimed to investigate its recovery by depolymerization, which could be of paramount importance in the circular economy of the FDCA. For this, a new polar aprotic solvent, methyl sesamol (MeSesamol), was used, allowing the facile depolymerization of PEF at room temperature with high monomer yields (up to 85 %), while the cosolvent MeSesamol was recycled with high efficiency (95–100 %) over five reaction cycles.

## Graphical abstract

Circularity of FDCA: An electrochemical transformation of 5‐hydroxymethylfurfural (HMF) to 2,5‐furandicarboxylic acid (FDCA) is reported using TEMPO in 0.1 M KOH solution. FDCA is the monomer of poly(ethylene furanoate), thus its recovery by depolymerization using a novel bio‐based co‐solvent MeSesamol is investigated.

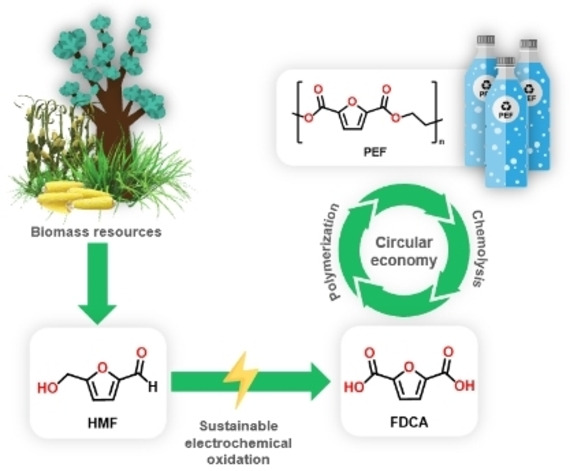

## 1 Introduction

Since the first industrial revolution, there has been a significant demand for fossil fuels to fulfil humankind′s needs.[^1^, ^2^] However, the depletion of fossil fuels and increased carbon emissions associated with their consumption have prompted the valorization of renewable alternatives to replace them. Biomass, as a non‐fossil‐based feedstock, presents a unique opportunity.[^3^, ^4^, ^5^, ^6^, ^7^] Some of the most important naturally produced chemicals are C_5_‐ and C_6_‐sugars, such as xylose, fructose, and glucose. These subunits typically form polysaccharides, such as starch, cellulose or chitin, which are widely available worldwide in nature, and can be converted into valuable renewable building blocks.[^8^, ^9^, ^10^, ^11^]

**Figure 1:**
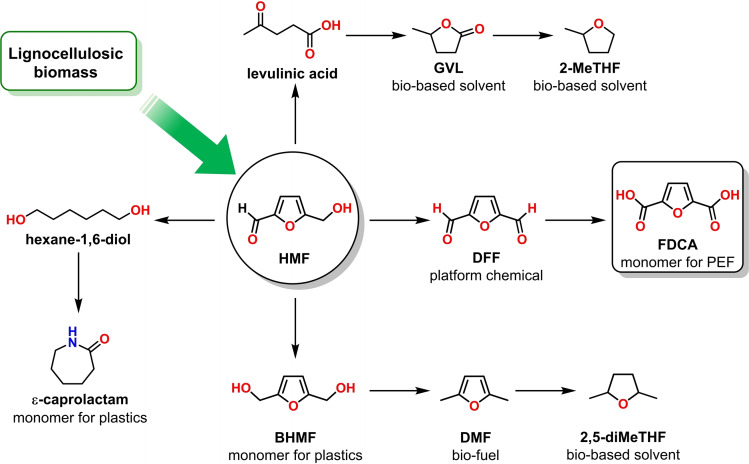
Versatile application of renewable HMF as a platform chemical.

HMF stands out as one of the most noteworthy biomass‐derived platform molecules.[^7^, ^12^, ^13^, ^14^, ^15^, ^16^] It can be transformed into many value‐added products, including levulinic acid that can be converted to γ‐valerolactone (GVL), 2,5‐dimethylfuran (DMF), 2,5‐ dimethyltetrahydrofuran, 2,5‐diformylfuran (DFF) or 2,5‐furandicarboxylic acid (Figure 1.). FDCA and its derivatives are emerging platform chemicals for the production of medicines and polymers.[^17^, ^18^] Unsurprisingly, FDCA has been ranked among the top‐value furan‐based molecules.^[12]^ Due to the high demand for FDCA, several electrochemical synthesis methods have been lately developed.[^19^, ^20^, ^21^, ^22^, ^23^, ^24^, ^25^, ^26^] HMF electrochemical oxidation reaction (HMFOR) to FDCA is a prospering technique that eliminates the consumption of O_2_ or other hazardous chemical oxidants to meet the requirements of green chemistry.^[20]^

The mechanism of HMFOR can be divided into direct and indirect oxidations. The direct oxidation of HMF to FDCA usually applies metal or metal oxide electrodes, such as Ni(OH)_2_,[^27^, ^28^] and Au(Pd)‐ZrO_x_[^29^, ^30^] under alkaline conditions. Constructing novel heterogeneous electrodes, such as Ni/Co,^[31]^ Ag–Co(OH)_2_ ^[32]^ and cation‐defective NiCo Prussian blue analog^[33]^ can result in improved electrocatalytic activity, thus further development in this area holds the potential for industrial application.

The indirect oxidation offers a homogeneous alternative where a mediator such as 2,2,6,6‐tetramethylpiperidine 1‐oxyl (TEMPO) is applied, facilitating the electron transfer between the substrate and the electrode without the participation of OH^−^. Thus, TEMPO‐mediated oxidation can be carried out in a weak base environment, and thus humin formation^[20]^ and Cannizzaro^[38]^ side‐reaction can be avoided. The non‐metallic catalysts could circumvent the drawbacks of metal catalysts, including high cost and product contamination by trace metals.^[19]^ Furthermore, TEMPO is often recyclable[^39^, ^40^] and has the advantage of avoiding the often time‐ and energy‐consuming preparation of metallic electrodes, as it can be used with cost‐effective, commercially available graphite electrodes. Cha and Choi showed the utilization of TEMPO as a mediator for HMFOR with nearly 100 % yield and Faradaic efficiency (FE) in a pH 9.2 borate buffer solution.^[34]^ Since then, further studies on the performance of TEMPO[^35^, ^37^, ^41^, ^42^, ^43^] and its derivative, such as 4‐acetamido‐TEMPO (ACT)[^35^, ^36^] and Hub‐TEMPO,^[40]^ for HMF (photo)‐electrooxidation have been demonstrated. The most prominent methods for the conversion of HMF into FDCA are summarized in Table 1. These methods apply constant potential that ensures the high selectivity of the product, however, requires a reference electrode and a potentiostat, which complicates the electrochemical setup. Thus, two‐electrode constant current electrolysis is often favored because of its simplicity, as it can be utilized with any low‐cost power supply without requiring a reference electrode.^[44]^

**Table 1:** Comparative analysis of the oxidation of HMF to FDCA using TEMPO derivatives.

Ref.	Anode–Cathode	Catalyst	Electrolyte	Mode	FDCA yield (%)
[34]	carbon felt – platinum	TEMPO 150 mol %	0.5 M borate buffer (pH 9.2)	constant potential (1.54 V)	99
[35]	carbon felt – platinum	TEMPO 40 mol %	0.5 M borate buffer (pH 10)	constant potential (1.4 V)	96.5
[35]	carbon felt – platinum	4‐acetamido‐TEMPO 40 mol %	0.5 M borate buffer (pH 10)	constant potential(1.4 V)	93.5
[36]	carbon felt – Ag/C	4‐acetamido‐TEMPO 10 mol %	0.5 M borate (pH 9.2)	constant potential (0.7 V)	98
[37]	BiVO_4_/CoPi‐30 (photoanode) – Pt wire	TEMPO 500 mol %	0.2 M borate buffer (pH 9.2)	constant potential (0.64 V)	88
**This work**	**Graphite – stainless steel**	**TEMPO** **5 mol %**	**0.1 M KOH** **(pH 13)**	**constant current (10 mA)**	**96**

One of the most prominent applications of FDCA is poly(ethylene‐furanoate), PEF, an emerging fully bioderived alternative to the fossil‐based poly(ethylene terephthalate), PET. Besides the demanding recycling and upcycling of PET waste,[^45^, ^46^, ^47^, ^48^, ^49^] the use of PEF offers a more sustainable solution. PEF has attracted attention mainly due to its similarity to PET, surpassing many of PET′s properties.^[50]^ Notably, PEF has an advantage in the packaging of carbonated drinks, as it is less permeable to CO_2_ than PET, preventing CO_2_ escape and oxygen penetration.^[51]^ Furthermore, PEF exhibits excellent thermal and mechanical behavior, with higher glass transition temperature,^[52]^ modulus and melting point compared to PET, rendering it mechanically more resilient. Beyond the mechanical and chemical properties of polymers, their end‐of‐life and recyclability are of paramount importance.[^53^, ^54^, ^55^, ^56^] Importantly, PEF is presumably recyclable using state‐of‐the‐art PET recycling techniques. In addition to mechanical recycling, chemical recycling of PEF has been marginally investigated, with researchers primarily focusing on enzymatic recycling,[^57^, ^58^, ^59^, ^60^, ^61^] and limited examples of common solvolytic routes,[^62^, ^63^, ^64^, ^65^] urging further investigation in this area.

In this work, we aimed to explore the potential of FDCA as a platform molecule. We demonstrated a TEMPO‐mediated electrocatalytic oxidation for the conversion of HMF into FDCA using the commercially available electrolysis cell, IKA ElectraSyn 2.0, equipped with inexpensive non‐precious‐metal‐based electrodes, graphite anode, and stainless‐steel cathode in constant current mode. Using a full factorial experimental design, we investigated electrochemical reaction conditions, such as the concentration of HMF and KOH, and the amount of TEMPO catalyst. Finally, to extend the possibilities of recycling the furan‐based PEF, we examined its depolymerization under basic conditions using MeSesamol^[66]^ as a cosolvent at room temperature, which may facilitate the circular economy of FDCA.

## 2 Results and Discussion

### 2.1 Electrocatalytic Oxidation of HMF to FDCA

**Scheme 1:**
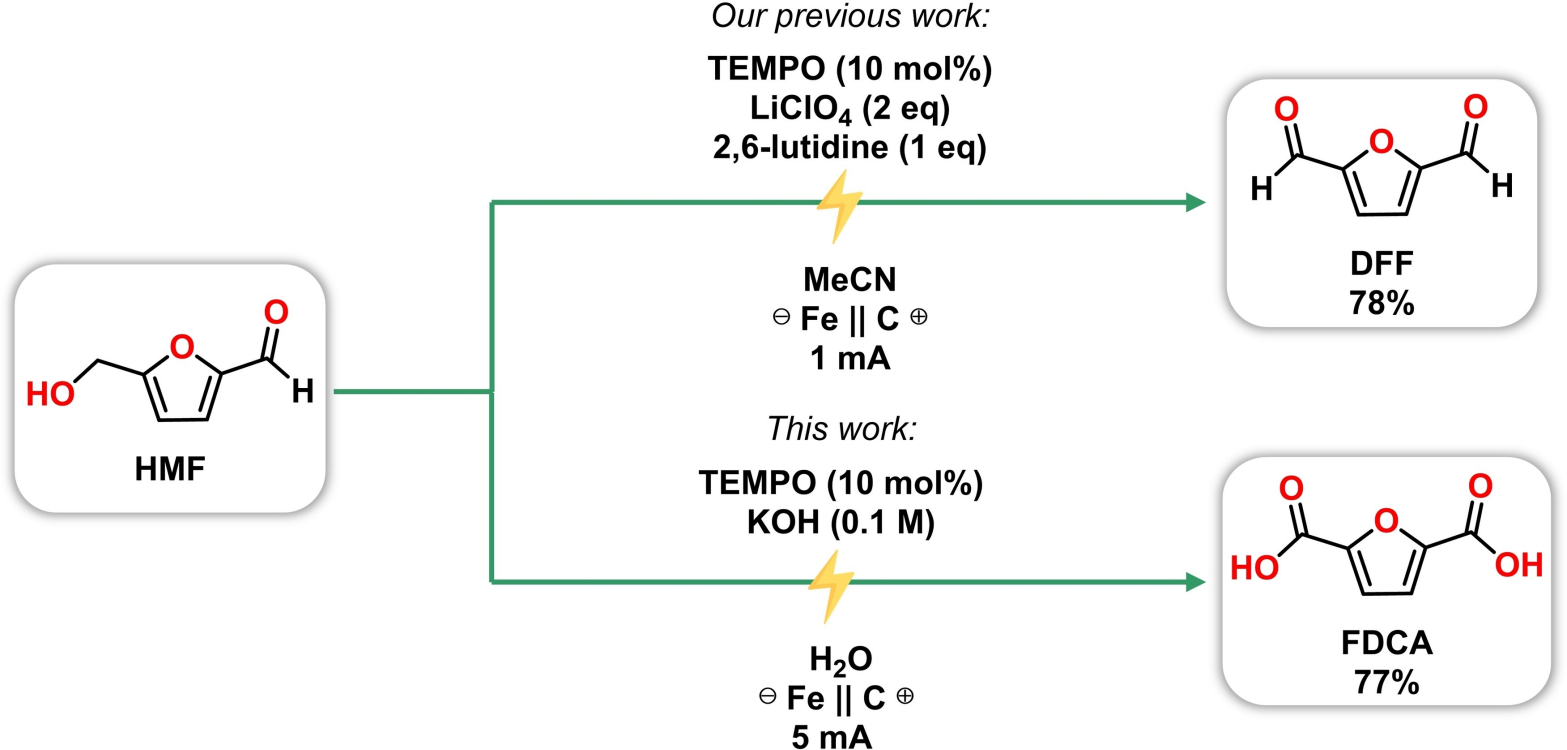
Fine‐tuning the electrochemical oxidation of HMF to gain FDCA.

Recently, our laboratory demonstrated size‐enlarged TEMPO‐mediated electrocatalytic oxidation of HMF into DFF with catalyst recycling facilitated by organic solvent nanofiltration.^[40]^ We showed that size‐enlarged TEMPO (Hub‐TEMPO) can be effectively recycled while it has similar activity to the native TEMPO in the electrooxidation reaction.^[40]^ Encouraged by these promising results, we embarked on a further exploration into the synthesis of FDCA from HMF by fine‐tuning the solvent, electrolyte, and base of the electrochemical oxidation. Based on the literature,^[19]^ the formation of FDCA is favorable under basic aqueous conditions. Consequently, we attempted the electrochemical oxidation by substituting the electrolyte (LiClO_4_) and base (2,6‐lutidine) with an aqueous solution of KOH (0.1 M), which serves as both electrolyte and base for the oxidation. We retrained the use of the cost‐effective graphite (anode) and stainless steel (cathode), however, we increased the current to 5 mA to reduce the reaction time. These adjustments enabled the production of FDCA with a good yield of 77 % (Scheme 1.). To optimize the HMFOR, three independent variables (i. e., the concentration of HMF and KOH, and the amount of TEMPO catalyst) were investigated, which factors were chosen based on preliminary investigations. We set up a 2^3^ full factorial experimental design and examined the importance of these three factors’ effects on yield. The lower and the upper levels of parameters were chosen from preliminary results. For instance, in the case of HMF concentration, at a higher concentration (1.0 M), we observed the reddening of the reaction and precipitation, presumably caused by the formation of furan‐based polymers called humin.^[67]^ It is well‐known that HMF is unstable and can quickly form humin.^[68]^ Moreover, a higher base concentration allows the intermolecular Cannizzaro reaction of HMF.[^69^, ^70^, ^71^] The experimental design matrix and the results are presented in Table 2.

**Table 2:** Levels of independent variables and the observed yield of FDCA in the experimental design space.

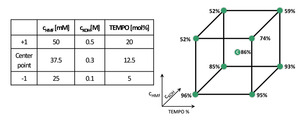				
Entry	*c_HMF_* ^[a]^ [mM]	*c_KOH_* ^[b]^ [M]	*TEMPO* ^[c]^ [mol %]	Yield^[d]^ [%]
1	25.0	0.1	5.0	96
2	25.0	0.5	5.0	85
3	25.0	0.1	20.0	95
4	25.0	0.5	20.0	93
5	50.0	0.1	5.0	52
6	50.0	0.5	5.0	52
7	50.0	0.1	20.0	74
8	50.0	0.5	20.0	59
9^[e]^	37.5	0.3	12.5	88
10^[e]^	37.5	0.3	12.5	80
11^[e]^	37.5	0.3	12.5	90

^[a]^ concentration of HMF ^[b]^ concentration of KOH ^[c]^ amount of TEMPO ^[d]^ FDCA yield was determined by HPLC. ^[e]^ center points of the design space.

The results of the analysis of variance (ANOVA) for the experimental design are shown in Table 3. Based on the ANOVA, only the concentration of HMF was found to be significant. A factor is considered significant if its *p*‐value is less than 0.05. The Pareto charts of effects in the case of FDCA yield as the dependent variable can be found in Figure S6 in the Supporting information.

**Table 3:** Analysis of variance (ANOVA) of FDCA yield.

Factor	Sum of squares	DF^[a]^	Mean square	*F*‐value	*p–*value	Significant
Curvature.	0.022827	1	0.022827	7.27015	0.114419	No
(1) KOH	0.009363	1	0.009363	2.98190	0.226342	No
(2) TEMPO	0.016132	1	0.016132	5.13771	0.151591	No
(3) HMF	0.219194	1	0.219194	69.80998	0.014024	Yes, p<0.05
1 by 2	0.000386	1	0.000386	0.12279	0.759490	No
1 by 3	0.000066	1	0.000066	0.02102	0.898012	No
2 by 3	0.006714	1	0.006714	2.13834	0.281171	No
1×2×3	0.006984	1	0.006984	2.22438	0.274357	No
Error	0.006280	2	0.003140			
Total SS	0.287946	10				

^[a]^ DF: degree of freedom.

Since only the concentration of HMF was significant, we examined the effect of its concentration to find the highest concentration that does not lead to the formation of by‐product. In this case, the concentration of both KOH and TEMPO was set to the lower level, 0.1 M and 5 mol %, respectively, considering both economic and environmental considerations. At the optimum concentration of HMF, the higher the concentration, the better the outcome, as this could increase the productivity of the reaction. The highest HMF concentration at which FDCA yield had not yet started to decrease was 31.25 mM (Table 4, entry 2). In addition, an attempt was made to increase the constant current for higher productivity, but lower yields were observed above 10 mA (see section 5 in Supporting information).

**Table 4:** Optimization of the concentration of HMF.

Entry	*c_HMF_* ^[a]^ [mM]	FDCA yield^[b]^ [%]
1	25	96
2	31.25	96
3	37.5	84
4	43.75	74
5	50	52

^[a]^ concentration of HMF ^[b]^ FDCA yield was determined by HPLC.

**Figure 2:**
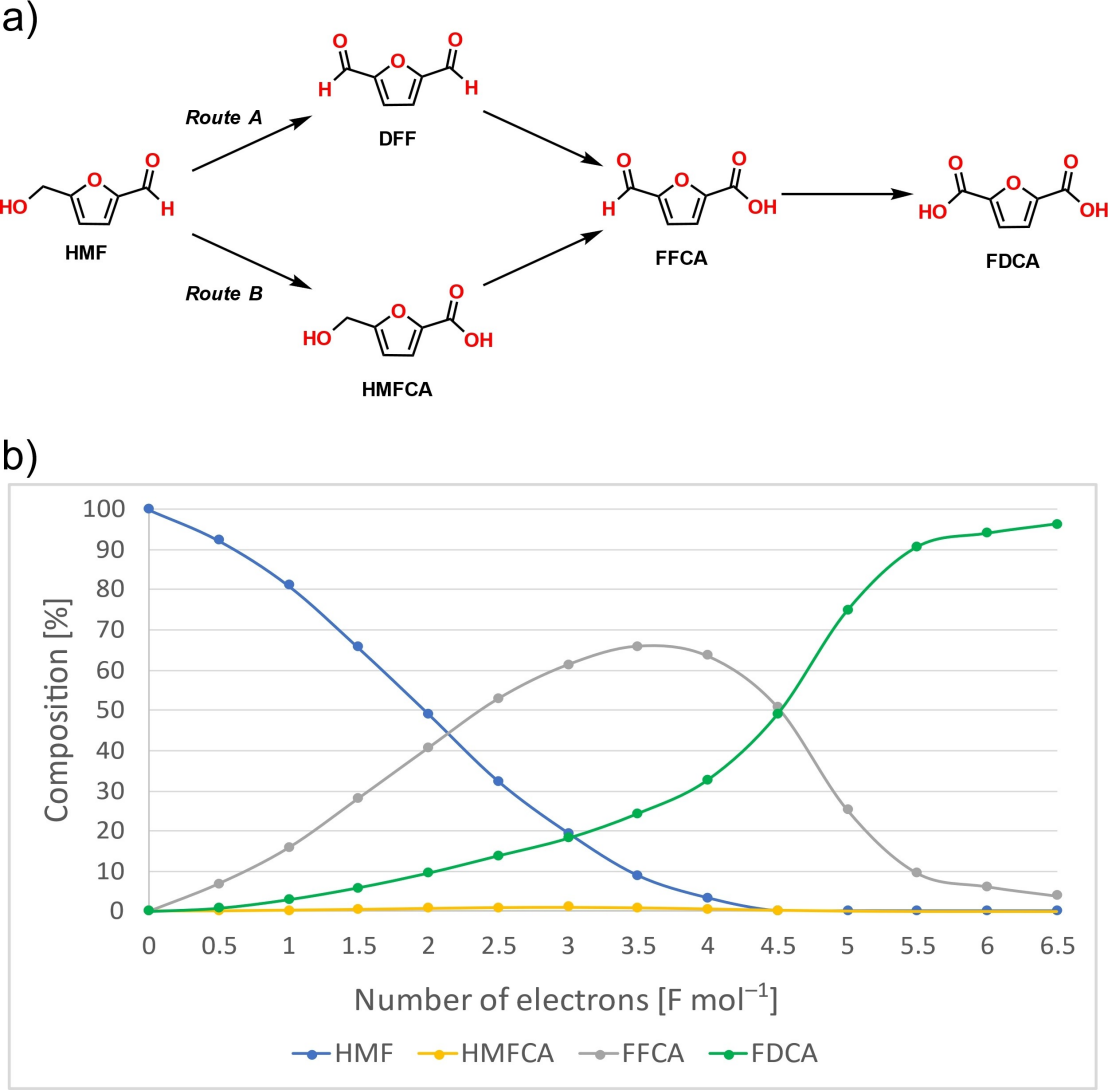
Possible reaction pathways for the electrochemical oxidation of HMF to FDCA (a) and the composition of the reaction mixture over the optimized electrochemical oxidation based on HPLC measurements (b).

To further understand the electrochemical oxidation of HMF using TEMPO, HPLC and NMR measurements were carried out. Based on the literature,^[19]^ the electrochemical oxidation of HMF to FDCA can occur in two possible pathways (Figure 2a). Through route A, the oxidation involves DFF as an intermediate, while *via* route B, the formation of 5‐hydroxymethyl‐2‐furancarboxylic acid (HMFCA) is observed as the first reaction intermediate by the oxidation of the aldehyde group. Then, both DFF and HMFCA are converted to 5‐formyl‐2‐furancarboxylic acid (FFCA), which is further oxidized to FDCA. These reaction pathways are pH‐dependent, with pathway A being the preferred pathway in non‐ strong alkaline environments (pH<13) and pathway B being the preferred pathway in strong alkaline environments (pH≥13), where the intermolecular Cannizzaro reaction must be considered. Our studies confirmed that the reaction follows the B pathway in our case, as no DFF formation was detected by either HPLC‐MS or NMR (Figure 2b). The HPLC results clearly show that the concentration of HMFCA is low throughout the electrochemical oxidation. This indicates that the conversion of HMFCA to FFCA is fast, and thus the rate‐determining step is the oxidation of FFCA to FDCA. Furthermore, the decomposition of FDCA was observed when the reaction was carried out beyond the theoretically necessary number of electrons (6 F mol^−1^ electron). This is presumably due to the fact that at the end of the electrooxidation, the concentration of the last intermediate, FFCA, in the reaction mixture is reduced to such an extent that the further oxidation of FDCA takes precedence. Finally, to prove that the reaction is TEMPO‐mediated, we investigated the possibility of direct oxidation of HMF without the use of TEMPO under the optimized conditions, but only negligible FDCA was observed.

### 2.2 Depolymerization of Poly(Ethylene Furanoate) to FDCA Under Basic Conditions

One of the most potential applications of furan dicarboxylic acid in the near future will be in the production of poly(ethylene furanoate), which could be an alternative to poly(ethylene terephthalate).^[19]^ The synthesis of polyethylene furanoate is well‐known in the literature,[50] but research on its recycling and depolymerization is still scarce. Previously, we reported that a new bio‐based polar aprotic solvent, MeSesamol, can help the depolymerization of PET and BPA‐PC due to its high solubilization power of numerous polymers.^[66]^

**Figure 3:**
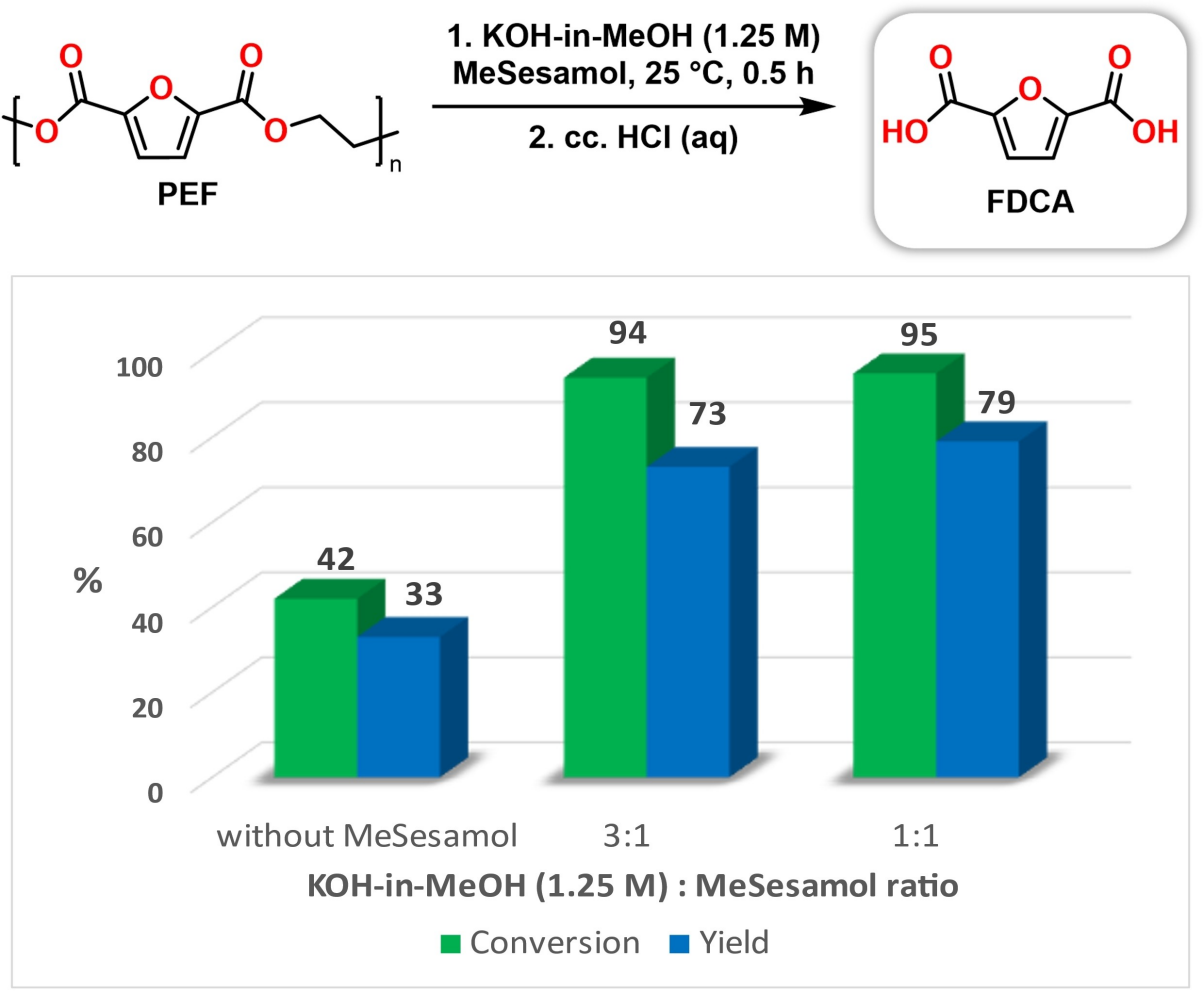
Investigation of the solubilization power of MeSesamol in the depolymerization of poly(ethylene furanoate).

Since PEF was found to be partially soluble in MeSesamol at room temperature, we envisioned that the application of MeSesamol as a cosolvent could also facilitate the depolymerization of PEF using KOH‐in‐MeOH hydrolysis.[^66^, ^72^, ^73^] To prove the positive effect of MeSesamol on depolymerization, the role of cosolvent was investigated in different proportions at room temperature. The results are shown in Figure 3. MeSesamol significantly increased PEF conversion (42 % vs. 94–95 %) at room temperature compared to depolymerization without MeSesamol. MeSesamol allows the reactions to be carried out effortlessly at ambient temperature with zero heating energy demands, which is particularly important from an environmental point of view and with today′s volatile energy prices. Furthermore, once the reaction was completed, water addition formed a biphasic system, allowing for the easy separation and recycling of the cosolvent. After the removal of volatile components from the aqueous phase and adjustment of pH to acidic (pH~1), the FDCA can be isolated by filtration, while MeSesamol can be reused in another reaction cycle.

**Figure 4:**
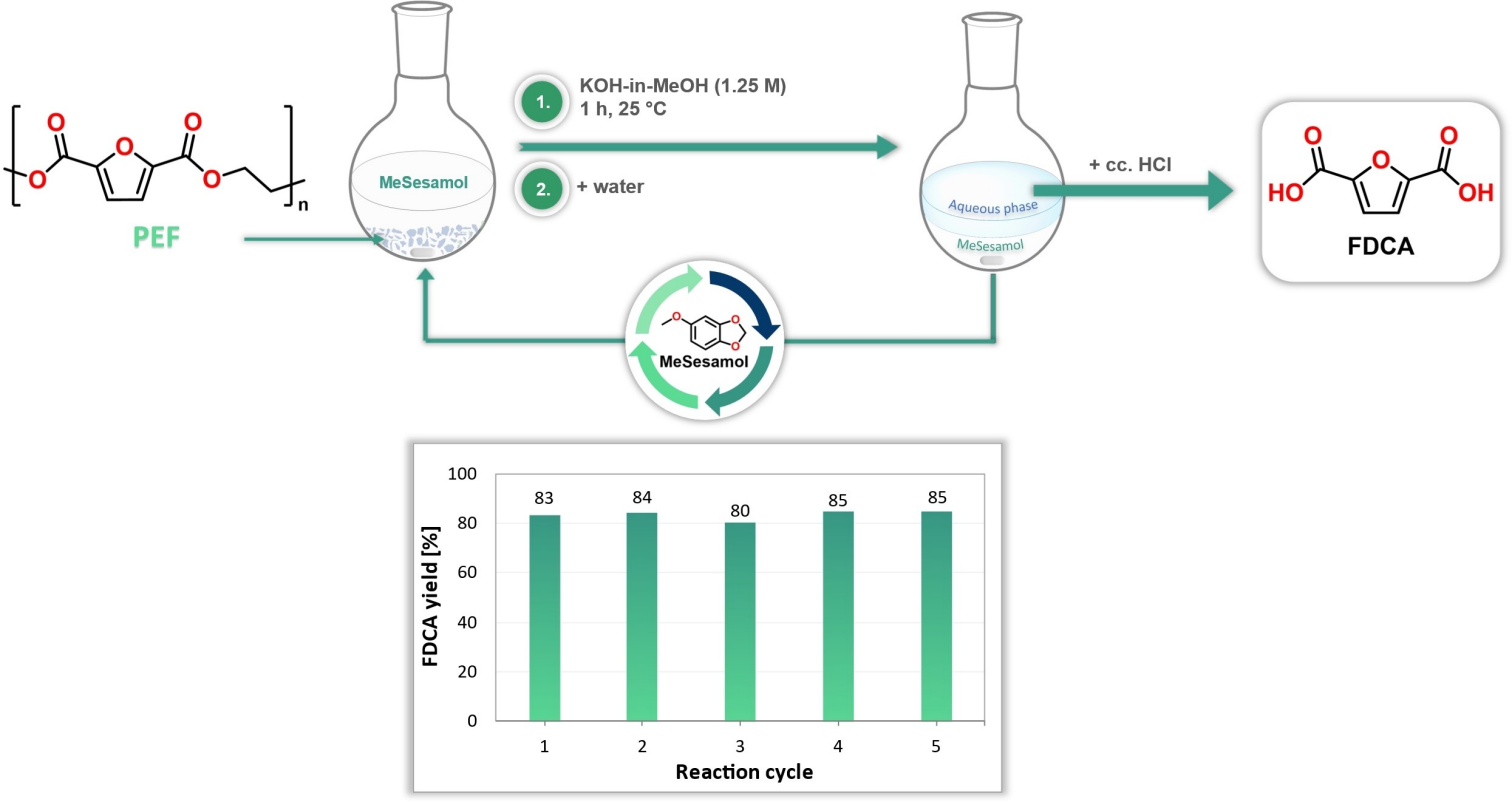
Depolymerization of poly(ethylene furanoate) by recycling MeSesamol cosolvent. After pH adjustment, the FDCA product was isolated from the aqueous phase by filtration, while MeSesamol was reused for a new reaction cycle.

We tested the recyclability of MeSesamol over 5 reaction cycles. We implemented the depolymerization reaction at room temperature for one hour to achieve full conversion of PEF. After the depolymerization was completed, FDCA was isolated from the aqueous phase by filtration and dried under vacuum. Over the five reaction cycles, the cosolvent MeSesamol was recycled with high efficiency (95–100 %), and high FDCA yields (up to 85 %) were achieved (Figure 4).

When comparing our method with other PEF depolymerization techniques, our approach stands out as one of the lowest‐temperature procedure for converting PEF back to its monomer (Table 5). The comparison greatly highlights the advantage of our method, as other methods often required high temperatures, longer reaction time and the application of pressure for depolymerization, which can make industrial feasibility challenging.

**Table 5:**
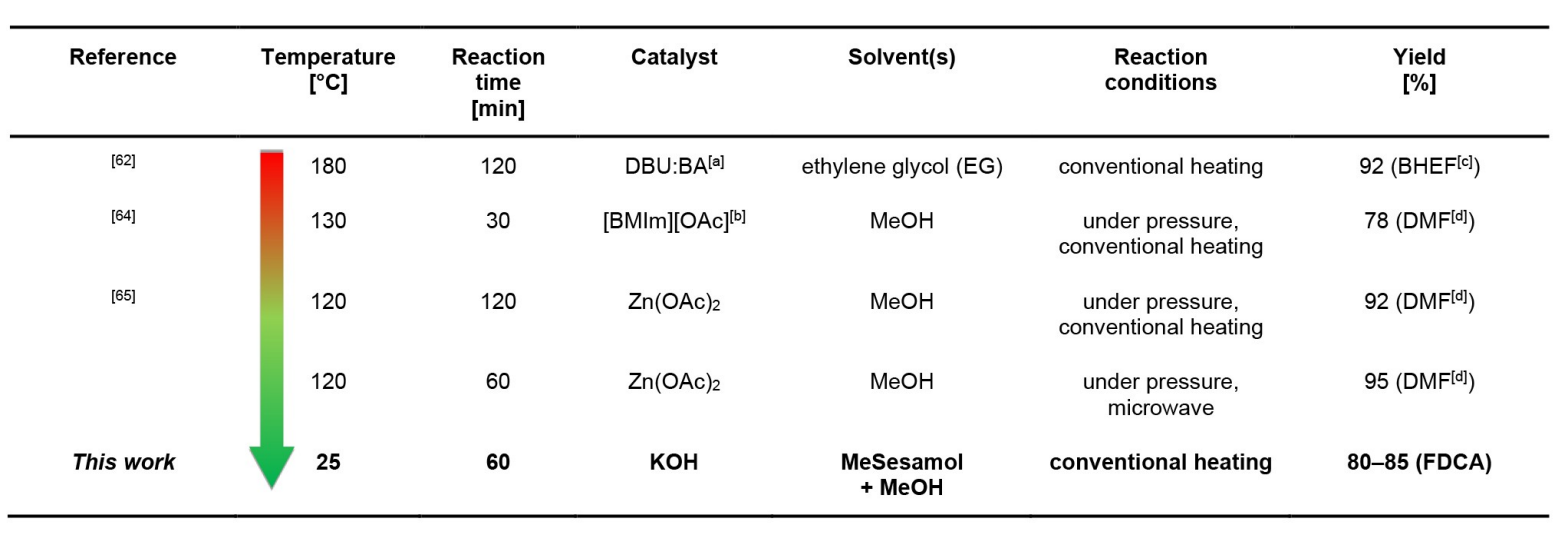
Comparison the conditions and effectiveness of our method with the reported PEF depolymerization procedures.

^[a]^ 1,8‐diazabicyclo[5.4.0]undec‐7‐ene:benzoic acid, ^[b]^ 1‐butyl‐3‐methylimidazolium acetate, ^[c]^ bis(hydroxyethyl) furanoate, ^[d]^ dimethyl furanoate.

**Figure 5:**
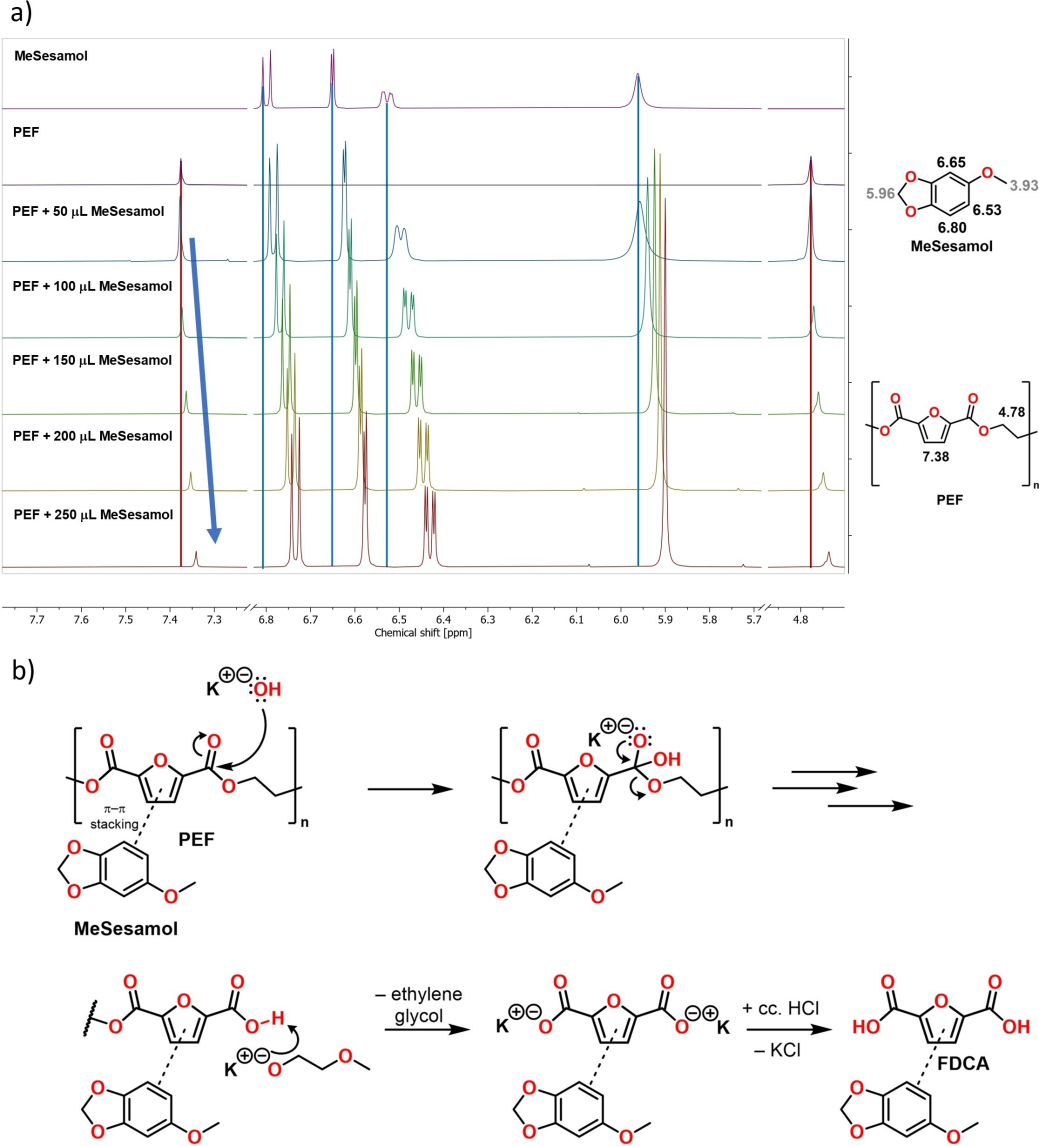
Changes in the ^1^H NMR chemical shift of PEF with increasing amounts of MeSesamol in trifluoroacetic acid–CDCl_3_ (1 : 1) mixture (a) and proposed reaction mechanism for MeSesamol co‐solvent‐assisted hydrolysis of PEF (b).

To better understand the role of MeSesamol in PEF degradation, we carried out ^1^H NMR studies. Previously, Le and co‐workers demonstrated that anisole is an effective cosolvent for PET depolymerization.^[74]^ Based on their studies, anisole helps PET degradation through intermolecular interactions. The addition of anisole to bis(2‐hydroxyethyl)terephthalate (BHET)–the monomer of PET–resulted in the downfield chemical shift of aromatic protons of BHET, suggesting π‐π interactions between the aromatic rings. Similar NMR analysis was conducted with different PEF–MeSesamol ratios, revealing a distinct change in chemical shift with the increasing molar ratio of MeSesamol, indicating π–π stacking of aromatic furan ring and MeSesamol (Figure 5a).

A comparable analysis was performed with the solution of FDCA, showing a similar shift of aromatic protons signals (see section 7.4 in Supporting information). Based on these experiments, MeSesamol not only increases the solubility of PEF in the reaction media but also assists the depolymerization by forming π‐π stacking with both PEF and the produced FDCA (Figure 5b).

Overall, the efficiency‐enhancing effects of MeSesamol as a cosolvent in depolymerization reactions are unquestionable, as the depolymerization of PEF at room temperature occurs twice as fast with cosolvent. Considering these results and previous studies, the subclass of aromatic cosolvents during the depolymerization of PET as well as PEF, is highly favored. Therefore, it is appropriate to investigate aromatic cosolvents further and explore their mechanisms in the near future.

Re‐polymerizing the recovered monomers into new polymers promotes the circular economy of these materials. Given that depolymerization reactions typically have yields below 100 %, additional starting material is required to compensate for this deficit. Our objective was to illustrate this holistic view of FDCA′s recyclability. Therefore, we combined electrochemically produced FDCA with the recovered FDCA obtained through depolymerization to re‐synthesize PEF. Following the literature procedure with minor modifications,^[75]^ we successfully obtained PEF. After the successful polymerization reaction, we closed the loop and depolymerized the gained PEF back to FDCA using the beforementioned MeSesamol cosolvent‐assisted procedure, achieving the same yield of 85 % as for commercial PEF (for more information see Section 6 in the Supporting information).

## 3 Conclusions

We have demonstrated a novel electrochemical oxidation of HMF to FDCA using an environmentally friendly alkaline aqueous electrolyte with high FDCA selectivity. Contrary to previously reported methods, we applied a constant current (10 mA) instead of constant potential, with only 5 mol % of TEMPO catalyst. A full factorial experimental design was conducted to optimize the electrochemical reaction, which showed that only the HMF concentration was significant among the examined factors. We have found that at high concentrations of HMF, electrochemical oxidation can be accompanied by the formation of humin, leading to undesired complex by‐products. Therefore, to avoid the humin formation, we have determined the maximum applicable HMF concentration. Under the optimized conditions, 96±3 % HPLC and 78±3 % preparative yields of FDCA were achieved.

Furthermore, we investigated the recoverability of FDCA from the bio‐based polymer PEF. We found that MeSesamol proved to be a recyclable cosolvent that can aid the depolymerization reaction of PEF with high monomer yields (up to 85 %) at room temperature, bringing us one step closer towards a circular economy of FDCA.

## 4 Experimental Section

### 4.1 General Information

The starting materials and reagents were purchased from commercially available sources (Merck, and VWR). The depolymerization reactions were performed using PEF granules from Zhengzhou Alfa Chemical Co., Ltd (China). The applied PEF granules were long strips with a size of 3–4 mm in length and a cross‐sectional diameter of 2–3 mm. Aquagel Porous Chromatography (APC) measurements were carried out using Acquity Advanced Polymer Chromatography System and Waters 2414 Refractive Index detector at 35 °C and columns at 30 °C. PEF has a number‐average molecular weight (M_n_) of 13 417 g mol^−1^, a weight‐average molecular weight (M_w_) of 16 221 g mol^−1^, and a polymer dispersity index of 1.21. Infrared (IR) spectra were recorded on a Bruker Alpha‐T Fourier‐transform IR (FTIR) spectrometer. Thin‐layer chromatography (TLC) was performed using silica gel 60 F_254_ (Merck) plates. The reactions were monitored by TLC and high‐performance liquid chromatography–mass spectrometry (HPLC–MS). The solvent ratios in the eluents are given in volume units (mL mL^−1^). Nuclear magnetic resonance (NMR) spectra were recorded on a Bruker DRX‐500 Avance spectrometer (at 500 and 126 MHz for the ^1^H and ^13^C spectra, respectively) or on a Bruker 300 Avance spectrometer (at 300 and 75.5 MHz for the ^1^H and ^13^C spectra, respectively) at specified temperatures. HPLC–MS was performed on an HPLC system using a Shimadzu LCMS‐2020 (Shimadzu Corp., Japan) device equipped with a Reprospher (Altmann Analytik Corp., Germany) 100 Å C18 (5 μm; 100×3 mm) column and a positive/negative double ion source with a quadrupole MS analyzer in the range 50–1000 m/z. The FDCA yields were determined by HPLC measurement that was performed on a Shimadzu 2020 (Shimadzu Corp., Japan) device equipped with an Ace^®^ Excel 3 C18‐AR (250×4.6 mm) column. Further details are available in the Supporting information. The electrochemical experiments were carried out by using an IKA ElectraSyn 2.0 potentiostat equipped with a single vial holder. The reactions were conducted in constant current mode, without a reference electrode. The ElectraSyn 2.0, electrodes and vials were purchased from IKA (www.ika.com). The electrodes were washed multiple times with water, and acetone, and were rubbed dry with tissue paper before each use. The dimensions (W×H×D) of the electrodes were 8×52.5×2 mm.

### 4.2 General Procedure for the Electrocatalytic Oxidation of HMF into FDCA

An ElectraSyn vial (5 mL) equipped with a magnetic stir bar was charged with HMF (19.8 mg, 0.157 mmol), TEMPO (1.2 mg, 0.00786 mmol, 5 mol %) and KOH aqueous solution (0.1 M, 5 mL). The ElectraSyn vial cap equipped with the anode (graphite) and cathode (stainless steel) was inserted into the mixture. The reaction mixture was electrolyzed at a constant current of 10 mA and a total charge of 6.0 F mol^−1^. In the case of factorial design, the FDCA yield was determined by HPLC (see section 2 in Supporting information). The preparative yield was also determined under optimized conditions. After the reaction was completed, the pH was adjusted to acidic (pH~1) with concentrated hydrochloric acid (500 μL), which resulted in the precipitation of FDCA. Then, the reaction mixture was filtered, and the FDCA was washed with distilled water (2×1 mL).

### 4.3 General Procedure of PEF Depolymerization with MeSesamol Recycling

The PEF granules (approximately 0.5 g), KOH‐in‐MeOH solution (1.25 M, 10 mL), and MeSesamol (10 mL) were charged into a round‐bottom flask. The purity of the applied potassium hydroxide flakes was 89.6 % (Lach‐ner Ltd., Czech Republic). The resulting heterogeneous mixture was stirred magnetically at 1000 rpm for 1 h at room temperature. Next, distilled water (10 mL) was added and the mixture was stirred until the white precipitate had completely dissolved (5 min). The resulting two phases were separated, and MeSesamol was reused in the following reaction cycle. The volatile components of the obtained homogeneous aqueous solution were removed in vacuo. Next, the pH was adjusted to acidic (pH~1) with concentrated hydrochloric acid (2 mL). After pH adjustment, the white precipitate was filtered, washed with distilled water (25 mL) and heptane (25 mL), and dried under a vacuum to obtain white powder of FDCA.

The isolated yield was calculated as

(1) $$Yield\left[\%\right]=\frac{m_{FDCA}}{m_{FDCA,\;theoretical}}\times 100\;\%$$

where $m_{FDCA}$ and $m_{FDCA,\;theoretical}$ refer to the actual and theoretical weight of FDCA, respectively. The theoretical weight of FDCA was calculated by the following Equation2

(2) $$m_{FDCA,\;theoretical}=\frac{m_{PEF}}{182}\times 156$$

where $m_{PEF}$ is the initial weight of PEF, 182 and 156 are the molecular weight of the PEF repeating unit and that of FDCA, respectively.

### 4.4 Experimental Design

The electrochemical oxidation was optimized using Statistica software (TIBCO Software Inc.) at 5 % significance level to perform an analysis of variance (ANOVA). Based on preliminary studies, the effects of three independent variables (i. e., the concentration of HMF and KOH, and the amount of TEMPO catalyst) on the FDCA yield (dependent variable) were investigated.

## Conflict of Interests

The authors declare no conflict of interest.

5

## Supporting information

As a service to our authors and readers, this journal provides supporting information supplied by the authors. Such materials are peer reviewed and may be re‐organized for online delivery, but are not copy‐edited or typeset. Technical support issues arising from supporting information (other than missing files) should be addressed to the authors.

Supporting Information

## Data Availability

The data that support the findings of this study are available in the supplementary material of this article.

## References

[1] C. Zou , Q. Zhao , G. Zhang , B. Xiong , Nat. Gas Ind. B 2016, 3, 1–11.

[2] S. Bilgen , Renew. Sustain. Energy Rev. 2014, 38, 890–902.

[3] G. W. Huber , S. Iborra , A. Corma , Chem. Rev. 2006, 106, 4044–4098. 16967928 10.1021/cr068360d

[4] A. Corma Canos , S. Iborra , A. Velty , Chem. Rev. 2007, 107, 2411–2502. 17535020 10.1021/cr050989d

[5] P. Gallezot , Chem. Soc. Rev. 2012, 41, 1538–1558. 21909591 10.1039/c1cs15147a

[6] Z. Sun , B. Fridrich , A. De Santi , S. Elangovan , K. Barta , Chem. Rev. 2018, 118, 614–678. 29337543 10.1021/acs.chemrev.7b00588 PMC5785760

[7] L. T. Mika , E. Cséfalvay , Á. Németh , Chem. Rev. 2018, 118, 505–613. 29155579 10.1021/acs.chemrev.7b00395

[8] C. Yang , G. Szekely , Adv. Membr. 2022, 2, 100041.

[9] R. Hardian , A. Alammar , T. Holtzl , G. Szekely , J. Memb. Sci. 2022, 658, 120743.

[10] F. Carolin C , T. Kamalesh , P. S. Kumar , R. V. Hemavathy , G. Rangasamy , Ind. Crops Prod. 2023, 203, 117221.

[11] J. Lv , X. Lv , M. Ma , D. H. Oh , Z. Jiang , X. Fu , Carbohydr. Polym. 2023, 299, 120142. 36876773 10.1016/j.carbpol.2022.120142

[12] J. J. Bozell , G. R. Petersen , Green Chem. 2010, 12, 539–554.

[13] A. A. Rosatella , S. P. Simeonov , R. F. M. Frade , C. A. M. Afonso , Green Chem. 2011, 13, 754–793.

[14] R. J. Van Putten , J. C. Van Der Waal , E. De Jong , C. B. Rasrendra , H. J. Heeres , J. G. De Vries , Chem. Rev. 2013, 113, 1499–1597. 23394139 10.1021/cr300182k

[15] T. Wang , M. W. Nolte , B. H. Shanks , Green Chem. 2014, 16, 548–572.

[16] C. Chen , M. Lv , H. Hu , L. Huai , B. Zhu , S. Fan , Q. Wang , J. Zhang , Adv. Mater. 2024, 36, 2311464. 10.1002/adma.202311464 38808666

[17] M. Sajid , X. Zhao , D. Liu , Green Chem. 2018, 20, 5427–5453.

[18] S. H. Park , C. Yang , N. Ayaril , G. Szekely , ACS Sustain. Chem. Eng. 2022, 10, 998–1007.

[19] Y. Yang , T. Mu , Green Chem. 2021, 23, 4228–4254.

[20] Y. Zhao , M. Cai , J. Xian , Y. Sun , G. Li , J. Mater. Chem. A 2021, 9, 20164–20183.

[21] K. Wadaugsorn , K. Y. Lin , A. Kaewchada , A. Jaree , RSC Adv. 2022, 12, 18084–18092. 35800325 10.1039/d2ra01976k PMC9208393

[22] T. Harhues , M. Padligur , F. Bertram , D. M. Roth , J. Linkhorst , A. Jupke , M. Wessling , R. Keller , ACS Sustain. Chem. Eng. 2023, 11, 8413–8419.

[23] H. Zhou , Y. Ren , B. Yao , Z. Li , M. Xu , L. Ma , X. Kong , L. Zheng , M. Shao , H. Duan , Nat. Commun. 2023 141 2023, 14, 1–12. 10.1038/s41467-023-41497-y PMC10497620 37699949

[24] L. Gidi , J. Amalraj , C. Tenreiro , G. Ramírez , RSC Adv. 2023, 13, 28307–28336. 37753399 10.1039/d3ra05623f PMC10519153

[25] P. Zhu , M. Shi , Z. Shen , X. Liao , Y. Chen , Chem. Sci. 2024, 15, 4723–4756. 38550706 10.1039/d4sc00546e PMC10967261

[26] W. H. Lie , Y. Yang , J. A. Yuwono , C. Tsounis , M. Zubair , J. Wright , L. Thomsen , P. Kumar , N. Bedford , J. Mater. Chem. A 2023, 11, 5527–5539.

[27] J. Zhang , W. Gong , H. Yin , D. Wang , Y. Zhang , H. Zhang , G. Wang , H. Zhao , ChemSusChem 2021, 14, 2935–2942. 34013575 10.1002/cssc.202100811

[28] L. Gouda , L. Sévery , T. Moehl , E. Mas-Marzá , P. Adams , F. Fabregat-Santiago , S. D. Tilley , Green Chem. 2021, 23, 8061–8068.

[29] Y. Zhang , Y. Cao , C. Yan , W. Liu , Y. Chen , W. Guan , F. Wang , Y. Liu , P. Huo , Chem. Eng. J. 2023, 459, 141644.

[30] Y. Zhang , Y. Liu , W. Guan , M. Cao , Y. Chen , P. Huo , Chinese Chem. Lett. 2024, 35, 108932.

[31] S. Fan , B. Zhu , X. Yu , Y. Gao , W. Xie , Y. Yang , J. Zhang , C. Chen , J. Energy Chem. 2024, 92, 1–7.

[32] P. Zhou , X. Lv , H. Huang , B. Cheng , H. Zhan , Y. Lu , T. Frauenheim , S. Wang , Y. Zou , Adv. Mater. 2024, 36, 2312402. 10.1002/adma.202312402 38328963

[33] H. Zhang , Q. Yang , S. Luo , Z. Liu , J. Huang , Y. Zheng , C. Hu , J. Zhang , X. Bao , P. Yuan , et al., ACS Catal. 2024, 14, 9565–9574.

[34] H. G. Cha , K. S. Choi , Nat. Chem. 2015, 7, 328–333. 25803471 10.1038/nchem.2194

[35] A. C. Cardiel , B. J. Taitt , K. S. Choi , ACS Sustain. Chem. Eng. 2019, 7, 11138–11149.

[36] X. H. Chadderdon , D. J. Chadderdon , T. Pfennig , B. H. Shanks , W. Li , Green Chem. 2019, 21, 6210–6219.

[37] D. J. Chadderdon , L. P. Wu , Z. A. McGraw , M. Panthani , W. Li , ChemElectroChem 2019, 6, 3387–3392.

[38] M. L. Krebs , A. Bodach , C. Wang , F. Schüth , Green Chem. 2023, 25, 1797–1802.

[39] H. A. Beejapur , Q. Zhang , K. Hu , L. Zhu , J. Wang , Z. Ye , ACS Catal. 2019, 9, 2777–2830.

[40] P. Kisszekelyi , R. Hardian , H. Vovusha , B. Chen , X. Zeng , U. Schwingenschlögl , J. Kupai , G. Szekely , ChemSusChem 2020, 13, 3127–3136. 32338429 10.1002/cssc.202000453 PMC7318667

[41] A. Kawde , M. Sayed , Q. Shi , J. Uhlig , T. Pullerits , R. Hatti-Kaul , Catalysts 2021, 11, 969.

[42] L. Zheng , P. Xu , Y. Zhao , Z. Bao , X. Luo , X. Shi , Q. Wu , H. Zheng , Appl. Catal. B Environ. 2023, 331, 122679.

[43] I. Carrai , R. Mazzaro , E. Bassan , G. Morselli , A. Piccioni , S. Grandi , S. Caramori , P. Ceroni , L. Pasquini , Sol. RRL 2023, 7, 2300205.

[44] M. Rafiee , M. N. Mayer , B. T. Punchihewa , M. R. Mumau , J. Org. Chem. 2021, 86, 15866–15874. 34546751 10.1021/acs.joc.1c01391

[45] S.-H. Park , A. Alammar , Z. Fulop , B. A. Pulido , S. P. Nunes , G. Szekely , Green Chem. 2021, 23, 1175–1184.

[46] F. Topuz , D. G. Oldal , G. Szekely , Ind. Eng. Chem. Res. 2022, 61, 9077–9086.

[47] Z. Fehér , J. Kiss , P. Kisszékelyi , J. Molnár , P. Huszthy , L. Kárpáti , J. Kupai , Green Chem. 2022, 24, 8447–8459.

[48] H. W. Lee , K. Yoo , L. Borchardt , J. G. Kim , Green Chem. 2024, 26, 2087–2093.

[49] P. Pereira , P. E. Savage , C. W. Pester , Green Chem. 2024, 26, 1964–1974.

[50] K. Loos , R. Zhang , I. Pereira , B. Agostinho , H. Hu , D. Maniar , N. Sbirrazzuoli , A. J. D. Silvestre , N. Guigo , A. F. Sousa , Front. Chem. 2020, 8, 542806. 10.3389/fchem.2020.00585 PMC7413100 32850625

[51] S. K. Burgess , G. B. Wenz , R. M. Kriegel , W. J. Koros , Polymer (Guildf). 2016, 98, 305–310.

[52] A. Codou , M. Moncel , J. G. Van Berkel , N. Guigo , N. Sbirrazzuoli , Phys. Chem. Chem. Phys. 2016, 18, 16647–16658. 27067510 10.1039/c6cp01227b

[53] M. Hong , E. Y. X. Chen , Green Chem. 2017, 19, 3692–3706.

[54] H. Jung , G. Shin , H. Kwak , L. T. Hao , J. Jegal , H. J. Kim , H. Jeon , J. Park , D. X. Oh , Chemosphere 2023, 320, 138089. 36754297 10.1016/j.chemosphere.2023.138089

[55] R. Hardian , A. Ghaffar , C. Shi , E. Y. X. Chen , G. Szekely , J. Membr. Sci. Lett. 2024, 4, 100067.

[56] Z. Fehér , R. Németh , J. Kiss , B. Balterer , K. Verebélyi , B. Iván , J. Kupai , Chem. Eng. J. 2024, 485, 149832.

[57] A. Pellis , K. Haernvall , C. M. Pichler , G. Ghazaryan , R. Breinbauer , G. M. Guebitz , J. Biotechnol. 2016, 235, 47–53. 26854948 10.1016/j.jbiotec.2016.02.006

[58] S. Weinberger , J. Canadell , F. Quartinello , B. Yeniad , A. Arias , A. Pellis , G. M. Guebitz , Catalysts 2017, 7, 318.

[59] S. Weinberger , K. Haernvall , D. Scaini , G. Ghazaryan , M. T. Zumstein , M. Sander , A. Pellis , G. M. Guebitz , Green Chem. 2017, 19, 5381–5384.

[60] H. P. Austin , M. D. Allen , B. S. Donohoe , N. A. Rorrer , F. L. Kearns , R. L. Silveira , B. C. Pollard , G. Dominick , R. Duman , K. El Omari , et al. Proc. Natl. Acad. Sci. U. S. A. 2018, 115, E4350–E4357. 29666242 10.1073/pnas.1718804115 PMC5948967

[61] F. Kawai , Y. Furushima , N. Mochizuki , N. Muraki , M. Yamashita , A. Iida , R. Mamoto , T. Tosha , R. Iizuka , S. Kitajima , AMB Express 2022, 12, 1–15. 36289098 10.1186/s13568-022-01474-y PMC9606173

[62] E. Gabirondo , B. Melendez-Rodriguez , C. Arnal , J. M. Lagaron , A. Martínez De Ilarduya , H. Sardon , S. Torres-Giner , Polym. Chem. 2021, 12, 1571–1580.

[63] L. Sipos, M. L. Olson (Furanix Technologies B. V.) *WO2012091573A9*, **2011**.

[64] X. Qu , G. Zhou , R. Wang , B. Yuan , M. Jiang , J. Tang , Green Chem. 2021, 23, 1871–1882.

[65] C. Alberti , K. Matthiesen , M. Wehrmeister , S. Bycinskij , S. Enthaler , ChemistrySelect 2021, 6, 7972–7975.

[66] G. Dargo , D. Kis , M. Gede , S. Kumar , J. Kupai , G. Szekely , Chem. Eng. J. 2023, 471, 144365.

[67] S. Liu , Y. Zhu , Y. Liao , H. Wang , Q. Liu , L. Ma , C. Wang , Appl. Energy Combust. Sci. 2022, 10, 100062.

[68] J. Heltzel , S. K. R. Patil , C. R. F. Lund , in Reaction Pathways and Mechanisms in Thermocatalytic Biomass Conversion II. (Eds: M. Schlaf , Z. Zhang ), Springer, Singapore, 2016, pp. 105–118.

[69] Z. Du , D. Yang , Q. Cao , J. Dai , R. Yang , X. Gu , F. Li , Bioresour. Bioprocess. 2023, 10, 1–17. 38647628 10.1186/s40643-023-00676-x PMC10991370

[70] S. Subbiah , S. P. Simeonov , J. M. S. S. Esperança , L. P. N. Rebelo , C. A. M. Afonso , Green Chem. 2013, 15, 2849–2853.

[71] H. Liu , N. Agrawal , A. Ganguly , Y. Chen , J. Lee , J. Yu , W. Huang , M. Mba Wright , M. J. Janik , W. Li , Energy Environ. Sci. 2022, 15, 4175–4189.

[72] L. C. Hu , A. Oku , E. Yamada , K. Tomari , Polym. J. 1997, 29, 708–712.

[73] J. J. Rubio Arias , W. Thielemans , Green Chem. 2021, 23, 9945–9956.

[74] N. H. Le , T. T. Van Ngoc , B. Shong , J. Cho , ACS Sustain. Chem. Eng. 2022, 10, 17261–17273.

[75] J. Stanley , Z. Terzopoulou , P. A. Klonos , A. Zamboulis , E. Xanthopoulou , S. Koltsakidis , D. Tzetzis , L. F. Zemljič , D. A. Lambropoulou , A. Kyritsis , et al., Polymers (Basel) 2023, 15, 2707. 37376353 10.3390/polym15122707 PMC10305139

