# Supplementary material for: Furandicarboxylic Acid (FDCA): Electrosynthesis and Its Facile Recovery From Polyethylene Furanoate (PEF) via Depolymerization
Source: ChemSusChem. 2024 Oct 28;18(3):e202401190. doi: 10.1002/cssc.202401190 (PMC11789986; doi:10.1002/cssc.202401190)
Supplement: Supplementary file 1 — Supporting Information [file CSSC-18-e202401190-s001.pdf]

ChemSusChem

Supporting Information

Furandicarboxylic Acid (FDCA): Electrosynthesis and Its Facile Recovery From Polyethylene Furanoate (PEF) via Depolymerization

Gyula Dargó, Dávid Kis, Amália Ráduly, Vajk Farkas, and József Kupai*

Supporting information

Furandicarboxylic Acid (FDCA): Electrosynthesis and Its Facile Recovery From Polyethylene Furanoate (PEF) *via* Depolymerization

Gyula Dargó[a], *Dávid Kis*[a], *Amália Ráduly*[a], *Vajk Farkas*[a,b], and *Jozsef Kupai*[a]*

[a] Department of Organic Chemistry and Technology, Budapest University of Technology and Economics, Műegyetem rakpart 3., H-1111 Budapest, Hungary

[b] Institute of Materials and Environmental Chemistry, Hungarian Research Network, Research Centre for Natural Sciences, Magyar tudósok körútja 2, Budapest 1117, Hungary

**Corresponding author:* kupai.jozsef@vbk.bme.hu

Contents

1. General 3

2. HPLC analysis of the electrochemical oxidation 4

   2.1. Method and HPLC chromatogram of the electrochemical oxidation 4

   2.2. HPLC calibration curve of FDCA 5

   2.3. HPLC calibration curve of HMF 5

   2.4. HPLC calibration curve of HMFCA 6

   2.5. HPLC calibration curve of FFCA 6

3. Experimental design using Statistica 7

4. Cyclic voltammetry of TEMPO 8

5. Examination of the effect of current strength on FDCA yield 9

6. General procedure for polymerization of FDCA to PEF and its consequent depolymerization back to FDCA 9

7. ^{1}H, ^{13}C, and DEPTQ NMR spectra 10

   7.1. FDCA produced in electrochemical oxidation 10

   7.2. FDCA produced in the depolymerization of PEF 11

   7.3. NMR spectra of recycled MeSesamol after the 5th cycle 12

   7.4. Investigation of molecular interactions between MeSesamol and FDCA 13

   7.5. NMR spectra of PEF gained by polymerization 14

1. General

The starting materials and reagents were purchased from commercially available sources (Merck, TCI Europe, and VWR). The depolymerization reactions were performed using PEF granules from Zhengzhou Alfa Chemical Co., Ltd (China). The applied PEF granules were long strips, similar to rice grains, with a size of 3–4 mm in length and a cross-sectional diameter of 2–3 mm. Aquagel Porous Chromatography (APC) measurements were carried out using Acquity Advanced Polymer Chromatography System and Waters 2414 Refractive Index detector at 35 °C and columns at 30 °C. The sample manager was used at 30 °C. Column manager container three columns in series, (4.6 × 150 mm): ACQUITY APCTM XT 200 Å 1.7 µm, ACQUITY APCTM XT 125 Å 2.5 µm, ACQUITY APCTM XT 45 Å 1.7 µm. The third-order calibration curve was used, with polystyrene standards (66000 Da, 21500 Da, 4920 Da, 2280 Da) employed for calibration. HPLC grade DCM was used as eluent (VWR). The flow rate was 0.5 mL min^{-1}. For the calculations of the relative molecular weights and relative-molecular-mass dispersity, Empower Chromatography Data System was used. The concentration of the sample was 5 mg mL^{-1}, and the volumes of injections were 50 µL. PEF has a number-average molecular weight (M_n) of 13 417 g mol^{-1}, a weight-average molecular weight (M_w) of 16 221 g mol^{-1}, and a polymer dispersity index of 1.21. The purity of the potassium hydroxide flakes was 89.6% (Lach-ner Ltd., Czech Republic). Infrared spectra were recorded on a Bruker Alpha-T FT-IR spectrometer. Thin-layer chromatography (TLC) was performed using silica gel 60 F_{254} (Merck) plates. The reactions were monitored by TLC and high-performance liquid chromatography–mass spectrometry (HPLC–MS). The solvent ratios in the eluents are given in volume units (mL mL^{-1}). Nuclear magnetic resonance (NMR) spectra were recorded on a Bruker DRX-500 Avance spectrometer (at 500 and 126 MHz for the ^1H and ^{13}C spectra, respectively) or on a Bruker 300 Avance spectrometer (at 300 and 75.5 MHz for the ^1H and ^{13}C spectra, respectively) at specified temperatures. HPLC–MS was performed on an HPLC system using a Shimadzu LCMS-2020 (Shimadzu Corp., Japan) device equipped with a Reprospher (Altmann Analytik Corp., Germany) 100 Å C18 (5 µm; 100 × 3 mm) column and a positive/negative double ion source with a quadrupole MS analyzer in the range 50–1000 m/z. The FDCA yields were determined by HPLC measurement that was performed on a Shimadzu 2020 (Shimadzu Corp., Japan) device equipped with an Ace® Excel 3 C18-AR (250 × 4.6 mm) column. All reaction

samples were taken immediately after completion of the reaction to avoid degradation of the FDCA. The optimization of electrochemical oxidation was conducted by using Statistica software (TIBCO Software Inc.) at 5% significance level to perform analysis of variance (ANOVA).

2. HPLC analysis of the electrochemical oxidation

2.1. Method and HPLC chromatogram of the electrochemical oxidation

The electrochemical oxidation was followed by HPLC using Ace® Excel 3 C18-AR (250 × 4.6 mm) column. The samples were eluted with gradient elution, using eluent A (0.1% HCOOH in H_2O) and eluent B (MeCN). The flow rate was set to 0.8 mL min^{-1}. The column temperature was kept at 40 °C, and the injection volume was 1 µL. The HPLC gradient program was the following:

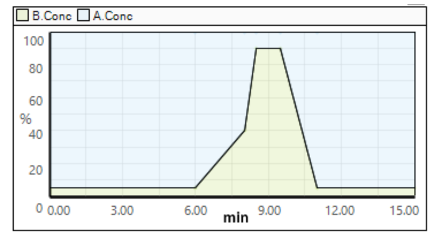

Time [min]	Eluent A [%] (0.1% HCOOH in H_2O)	Eluent B [%] (MeCN)
0–6	95	5
8	60	40
8.5	10	90
8.5–9.50	10	90
11	95	5
11–15	95	5

==== Shimadzu LabSolutions Multi-Chromatogram ====

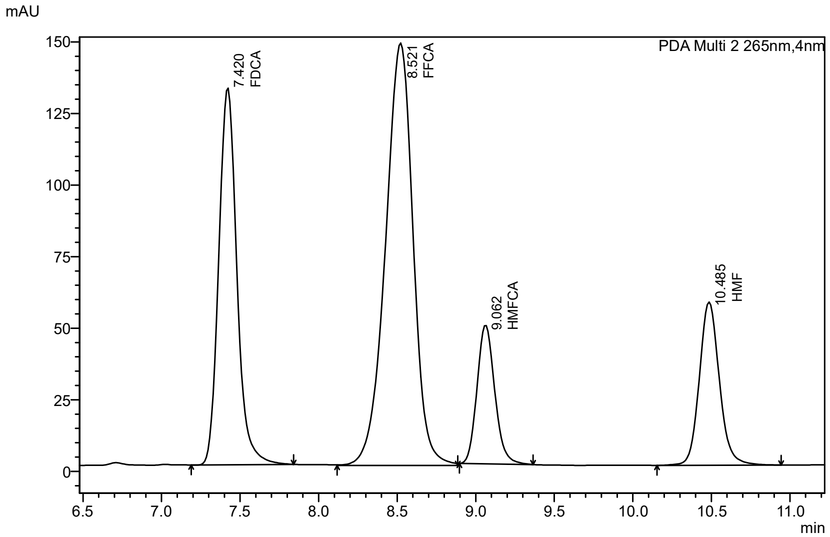

***Figure S1** A chromatogram at a selected state Q= 4 F mol^{-1}) of electrochemical oxidation reaction visualizing retention times of the components*

2.2. HPLC calibration curve of FDCA

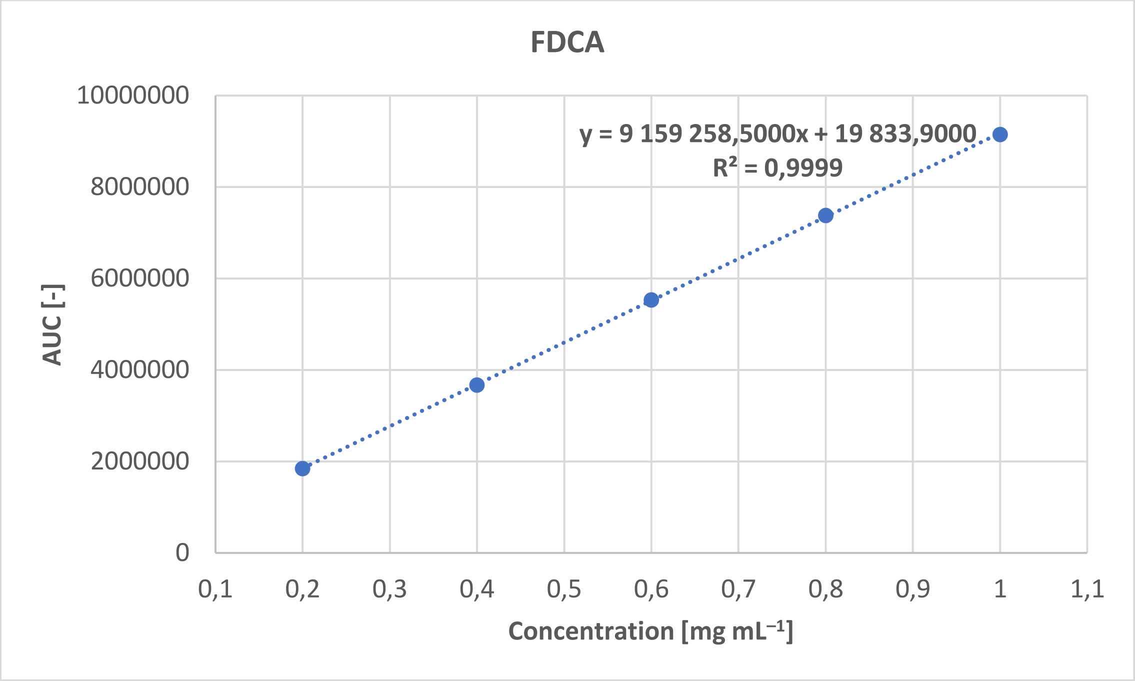

Figure S2 *The calibration curve of FDCA using Ace® Excel 3 C18-AR column*

2.3. HPLC calibration curve of HMF

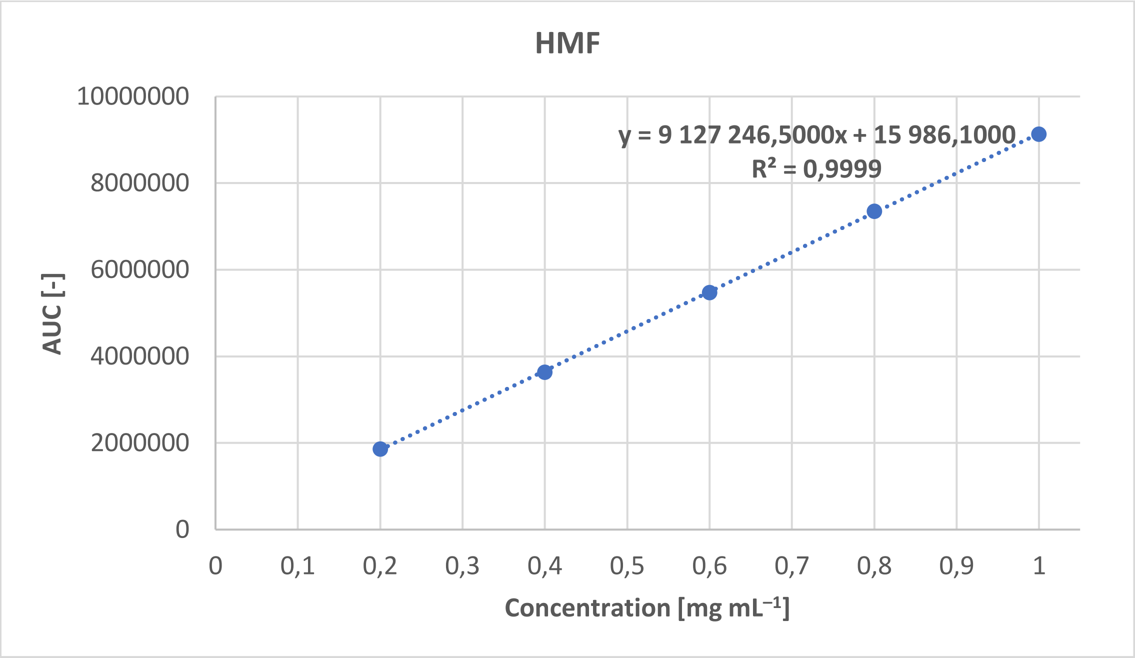

Figure S3 *The calibration curve of HMF using Ace® Excel 3 C18-AR column*

2.4. HPLC calibration curve of HMFCA

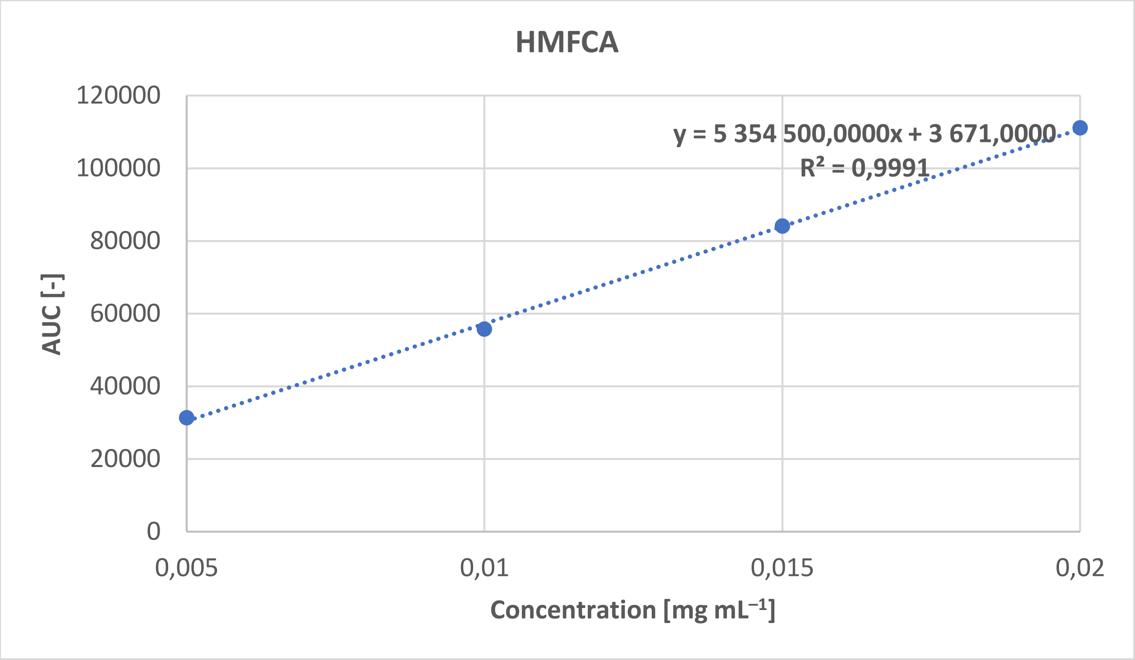

Figure S4 *The calibration curve of HMFCA using Ace® Excel 3 C18-AR column*

2.5. HPLC calibration curve of FFCA

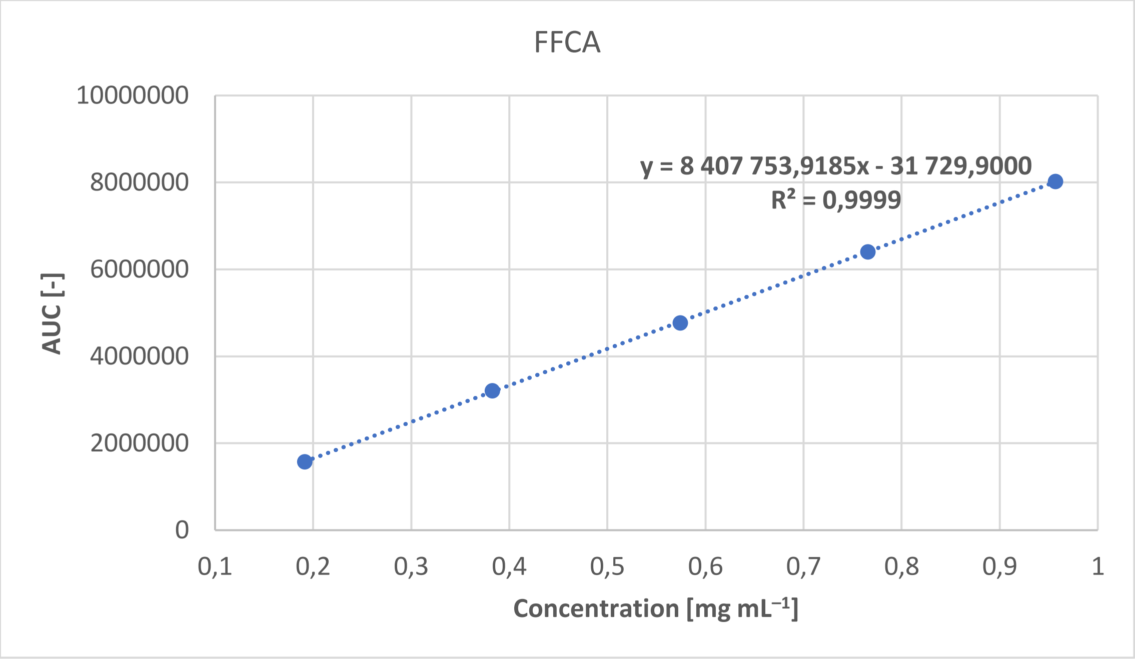

Figure S5 *The calibration curve of FFCA using Ace® Excel 3 C18-AR column*

3. Experimental design using Statistica

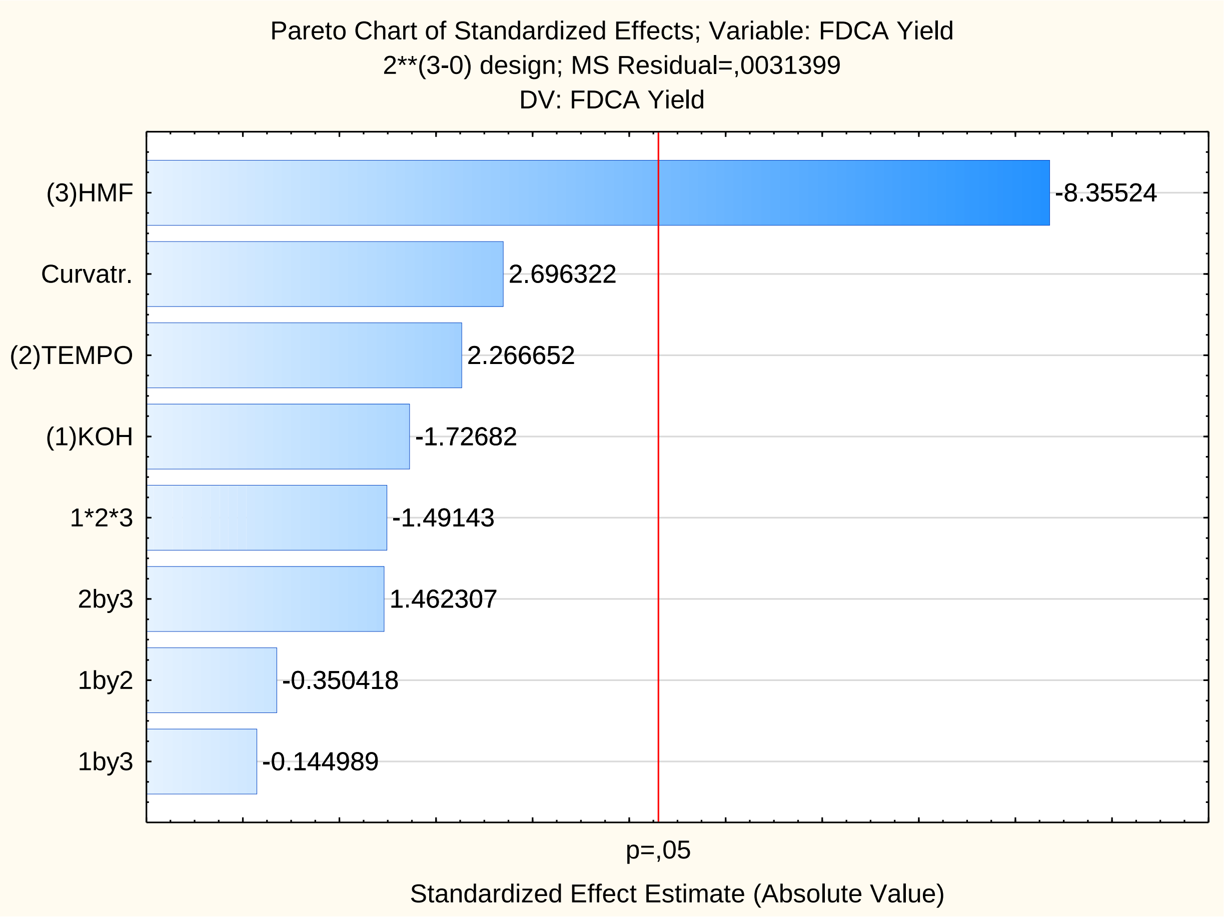

Figure S6 *Pareto chart of standardized effects in the case of FDCA yield as a dependent variable*

Effect Estimates; Var.:FDCA Yield; R-sqr=,97819; Adj:,89096 (Design: 2**(3-0) design ([No active dataset]) in FDCA kisterv)
2**(3-0) design; MS Residual=,0031399
DV: FDCA Yield

Factor	Effect	Std.Err.	t(2)	p	-95,% Cnf.Limt	+95,% Cnf.Limt	Coeff.	Std.Err. Coeff.	-95,% Cnf.Limt	+95,% Cnf.Limt
Mean/Interc.	0.758104	0.019811	38.26642	0.000682	0.672863	0.843345	0.758104	0.019811	0.672863	0.843345
Curvatr.	0.204573	0.075871	2.69632	0.114419	-0.121874	0.531020	0.102287	0.037936	-0.060937	0.265510
(1)KOH	-0.068421	0.039622	-1.72682	0.226342	-0.238902	0.102061	-0.034210	0.019811	-0.119451	0.051030
(2)TEMPO	0.089810	0.039622	2.26665	0.151591	-0.080671	0.260292	0.044905	0.019811	-0.040336	0.130146
(3)HMF	-0.331055	0.039622	-8.35524	0.014024	-0.501536	-0.160573	-0.165527	0.019811	-0.250768	-0.080287
1 by 2	-0.013884	0.039622	-0.35042	0.759490	-0.184366	0.156597	-0.006942	0.019811	-0.092183	0.078299
1 by 3	-0.005745	0.039622	-0.14499	0.898012	-0.176226	0.164737	-0.002872	0.019811	-0.088113	0.082368
2 by 3	0.057940	0.039622	1.46231	0.281171	-0.112541	0.228422	0.028970	0.019811	-0.056271	0.114211
1*2*3	-0.059094	0.039622	-1.49143	0.274357	-0.229576	0.111387	-0.029547	0.019811	-0.114788	0.055694

Figure S7 *Analysis of variance for the electrochemical oxidation. Only HMF concentration in red is proved to be statistically significant.*

4. Cyclic voltammetry of TEMPO

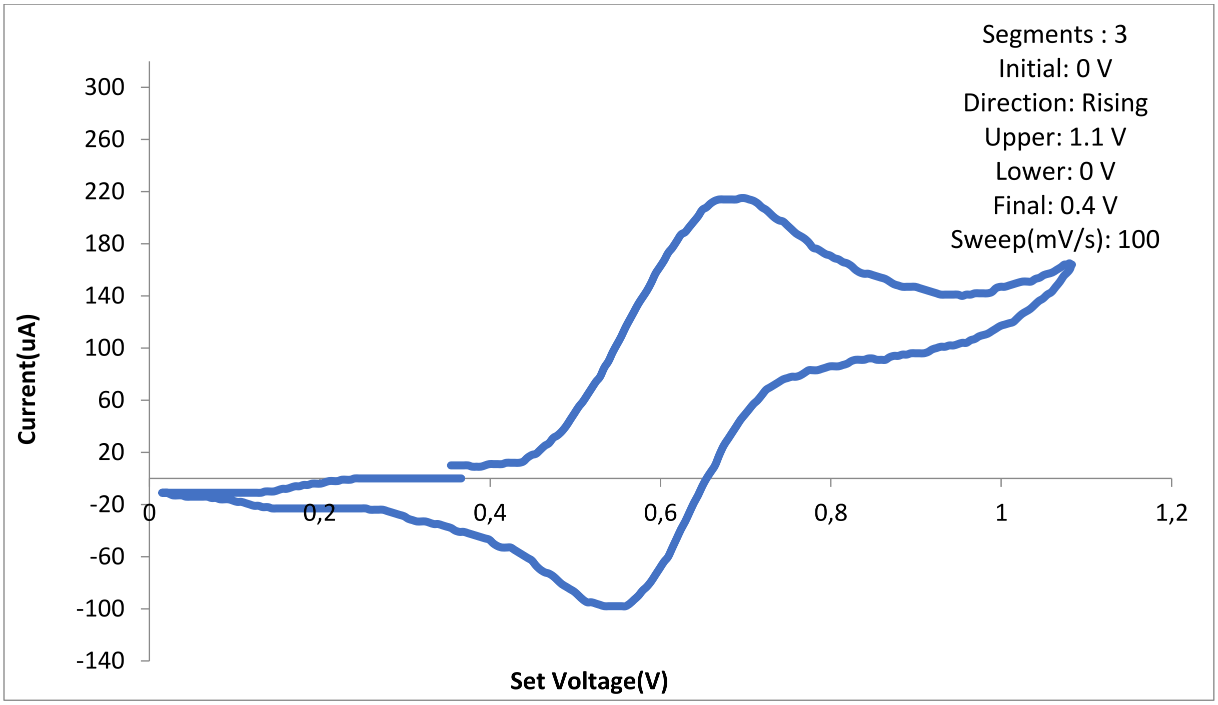

Figure S8 *CVs of TEMPO in 0.1 M KOH (aq). ElectraSyn CV package electrodes: glassy carbon (WE) and Pt (CE), with Ag/AgCl reference electrode*

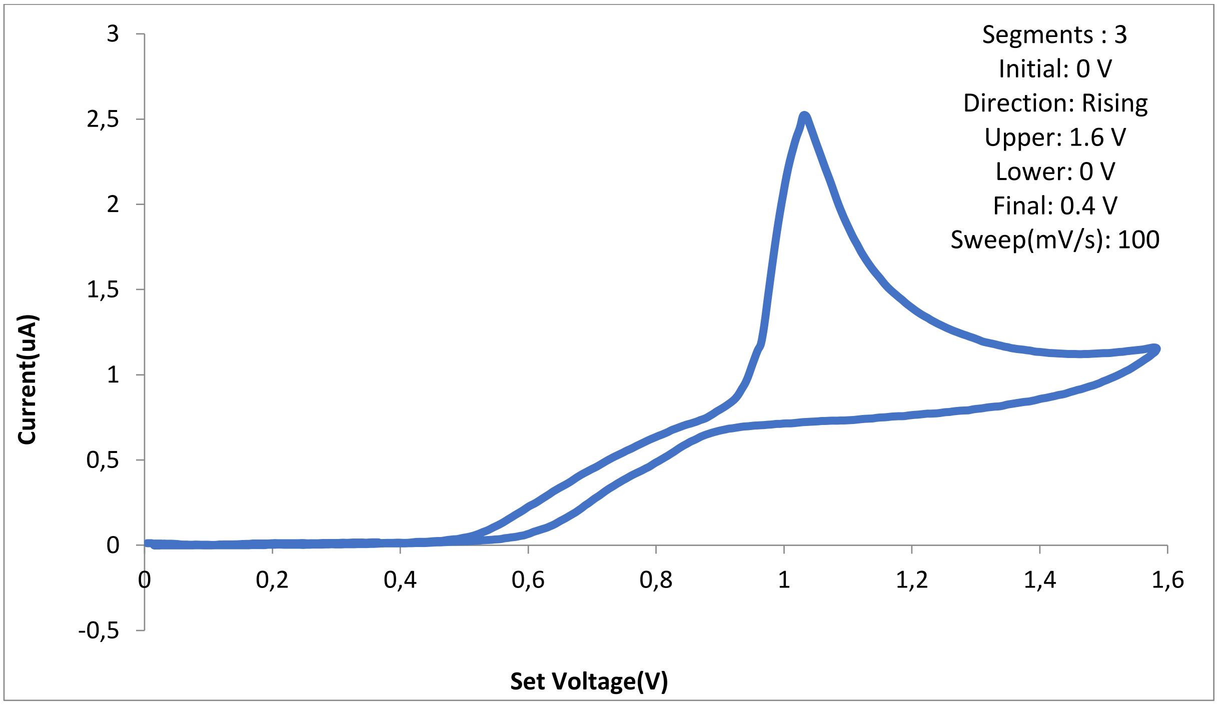

Figure S9 *CVs of the mixture of TEMPO and HMF in 0.1 M KOH (aq). ElectraSyn CV package electrodes: glassy carbon (WE) and Pt (CE), with Ag/AgCl reference electrode*

5. Examination of the effect of current strength on FDCA yield

An attempt was made to increase the constant current for achieving higher productivity, but a decrease in yield was observed.

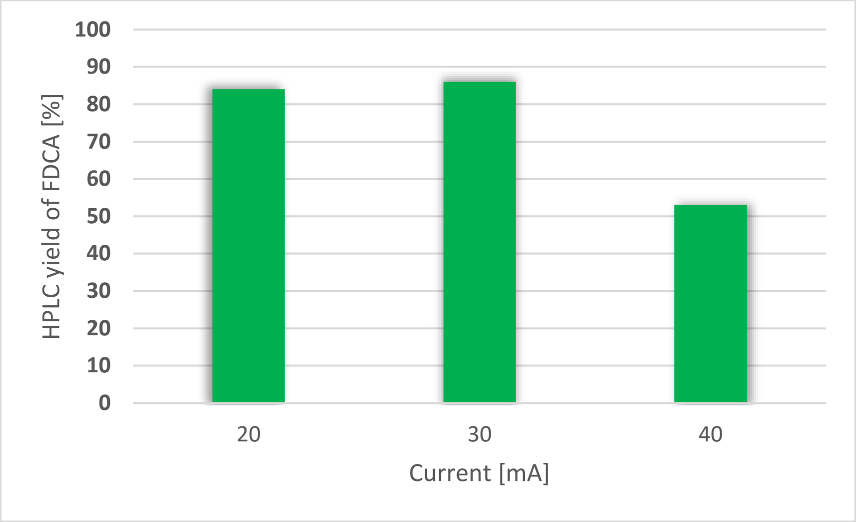

Figure S10 *The effect of constant current on FDCA yield*

6. General procedure for polymerization of FDCA to PEF and its consequent depolymerization back to FDCA

For the synthesis of PEF, we followed the literature procedure[1] with minor modifications. In the first esterification step, FDCA (2 g, 12.8 mmol, 90% from depolymerization reaction, 10% from electrosynthesis), EG (1.51 mL, 1.67 g, 26.9 mmol) and antimony trioxide (Sb_2O_3, 300 ppm) were measured into a flask. Initially, the flask was evacuated and filled with argon three times to completely remove the air. The reaction mixture was then heated at 170 °C for 30 min and 190–200 °C for 1 h under argon flow with a stirring speed of 200 rpm. During the first 1–1.5 hours, water distillation occurred. After 1.5 h of heating, the pressure was progressively decreased to 10 Pa over 15 min. Additionally, the temperature was gradually increased to 240–250 °C, and heating was continued for 4 hours. The reaction was quenched in ice water and the product was dissolved in pure HFIP (15 mL), then precipitated in THF (50 mL), filtrated, washed with MeOH

---

[1] J. Stanley, Z. Terzopoulou, P. A. Klonos, A. Zamboulis, E. Xanthopoulou, S. Koltsakidis, D. Tzetzis, L. F. Zemljič, D. A. Lambropoulou, A. Kyritsis, et al., *Polymers* **2023**, *15*, 2707.

(25 mL) and dried, which yielded off-white solid PEF (1.4 g, 60%). Then we followed the general procedure for PEF depolymerization to obtain FDCA again, achieving a good yield of 85% (Scheme S1.).

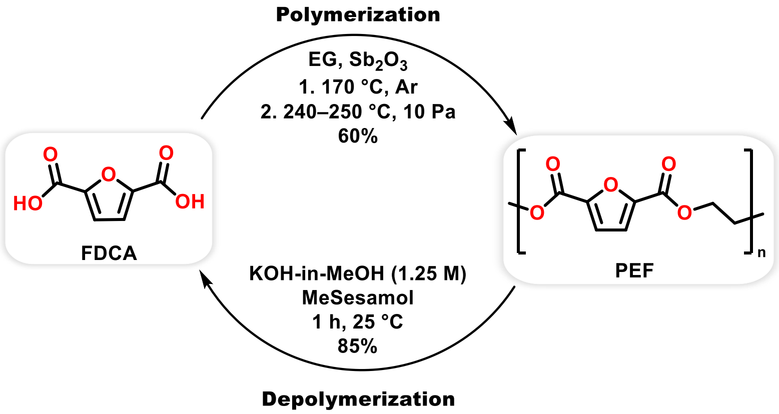

Scheme S1 *Circularity of FDCA in PEF recycling: direct polymerization with ethylene glycol and its recovery via cosolvent-assisted depolymerization*

7. ^{1}H, ^{13}C, and DEPTQ NMR spectra

7.1. FDCA produced in electrochemical oxidation

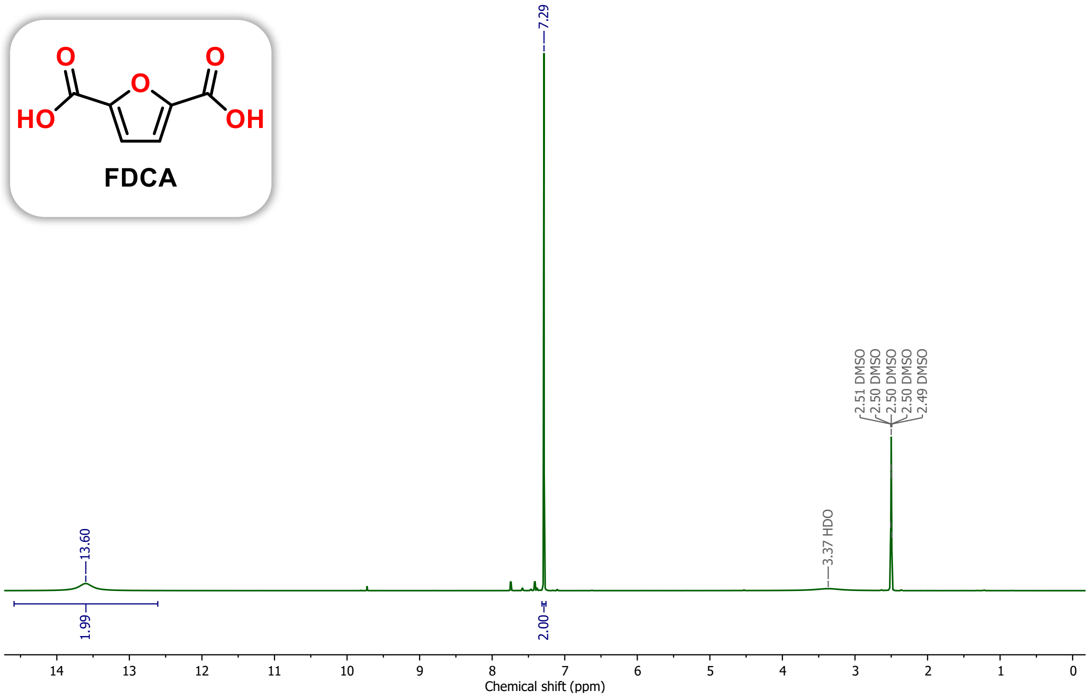

Figure S11 *^{1}H NMR spectrum of FDCA isolated by filtration*

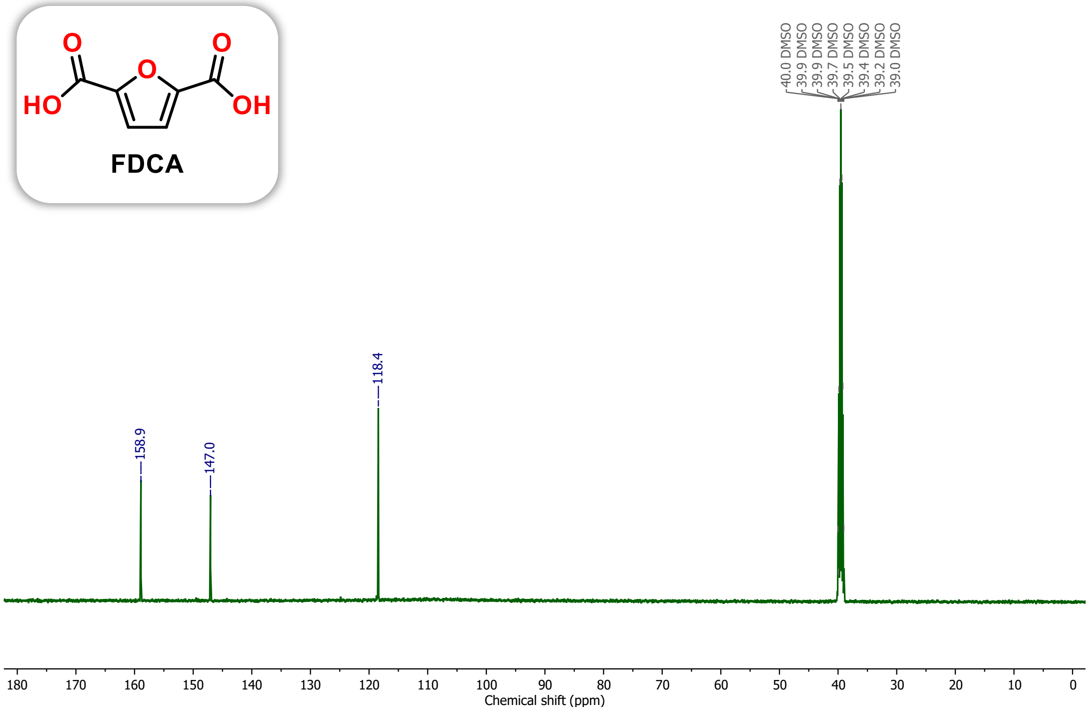

Figure S12 *^{13}C NMR spectrum of FDCA isolated by filtration*

7.2. FDCA produced in the depolymerization of PEF

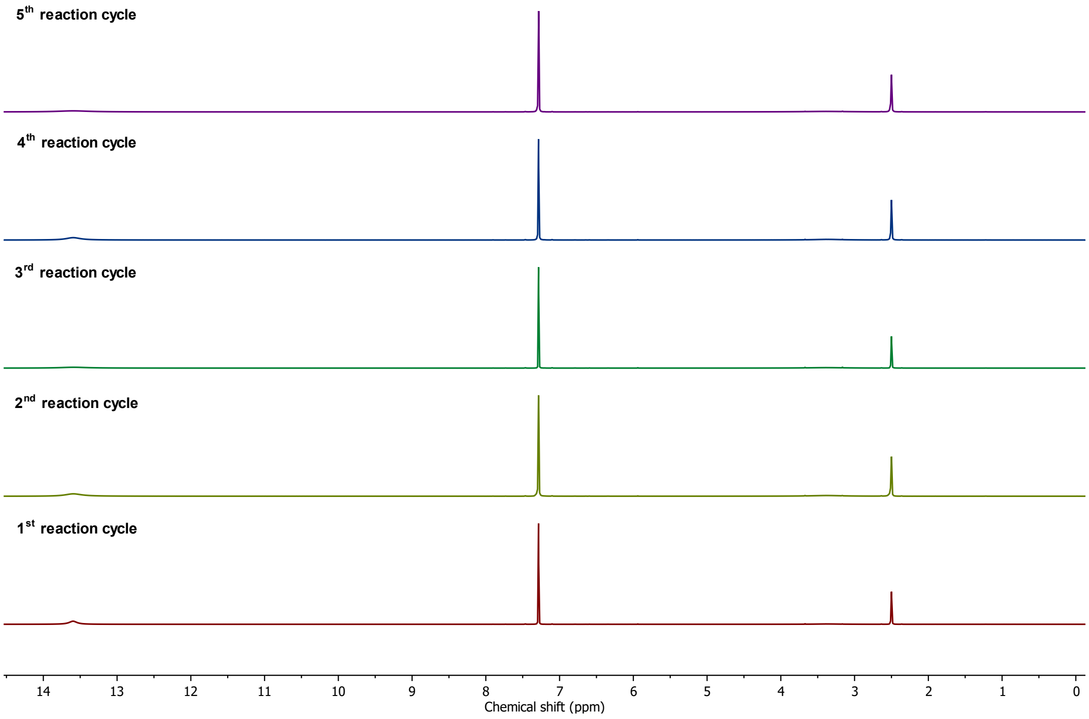

Figure S13 *^{1}H NMR spectrum of FDCA in DMSO-d_6 in different reaction cycles*

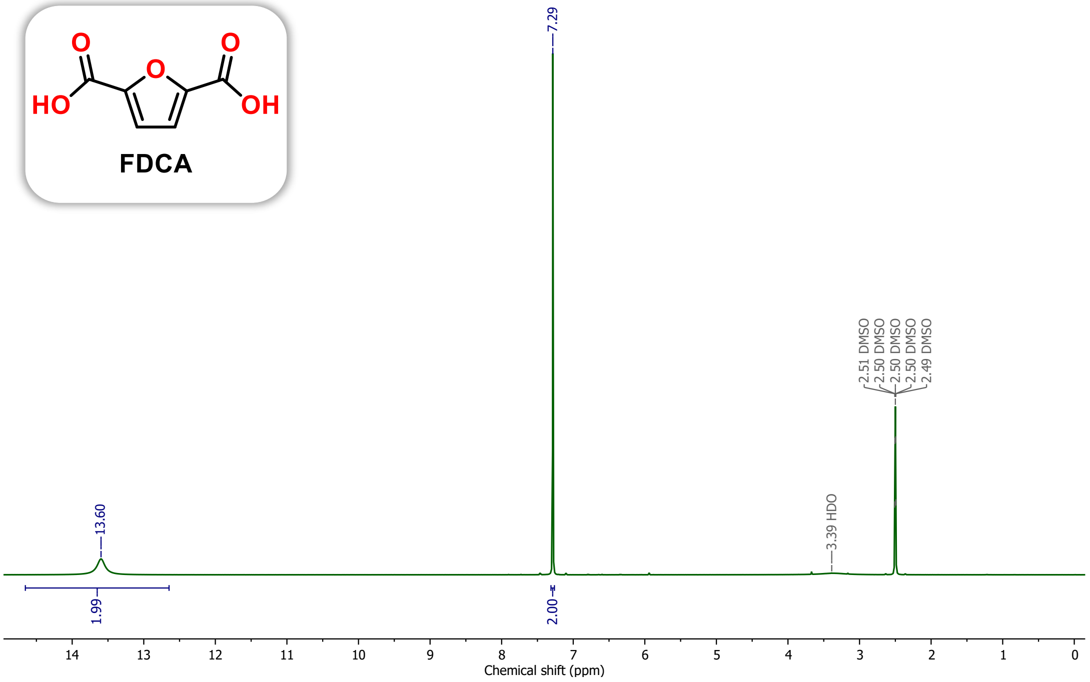

Figure S14 *^{1}H NMR spectrum of FDCA in DMSO-d$_6$ in the first reaction cycle*

7.3. NMR spectra of recycled MeSesamol after the 5th cycle

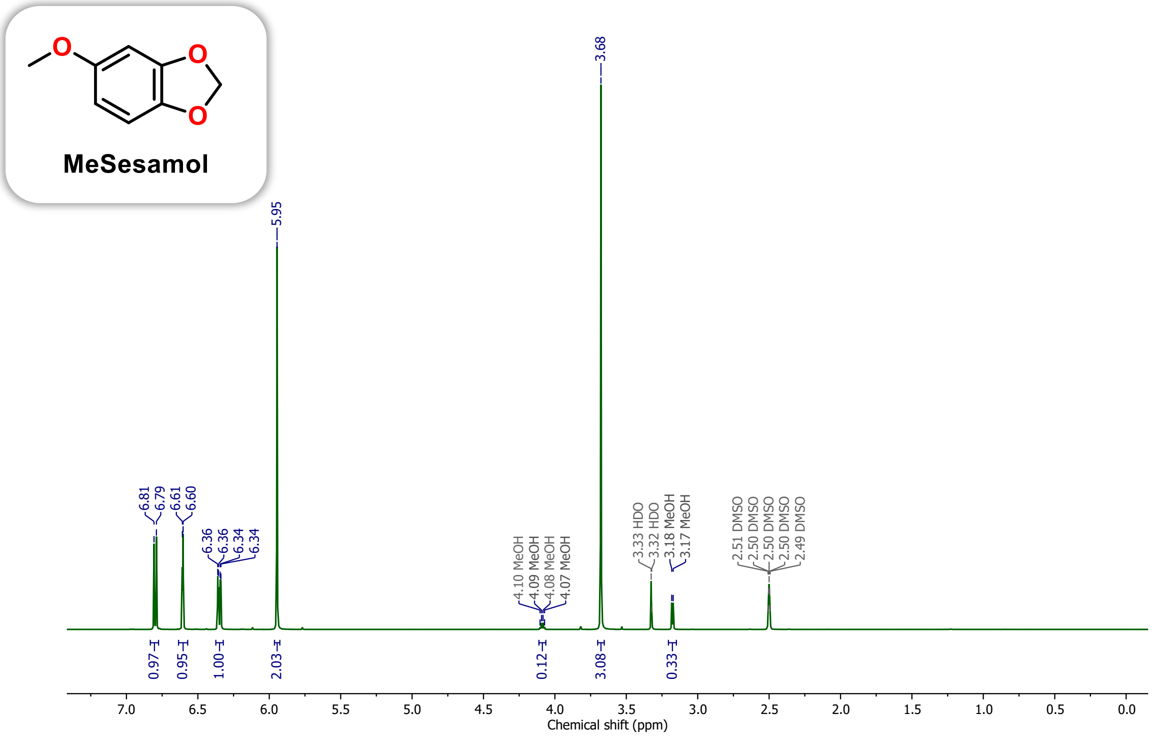

Figure S15 *^{1}H NMR spectrum of recycled MeSesamol in DMSO-d$_6$*

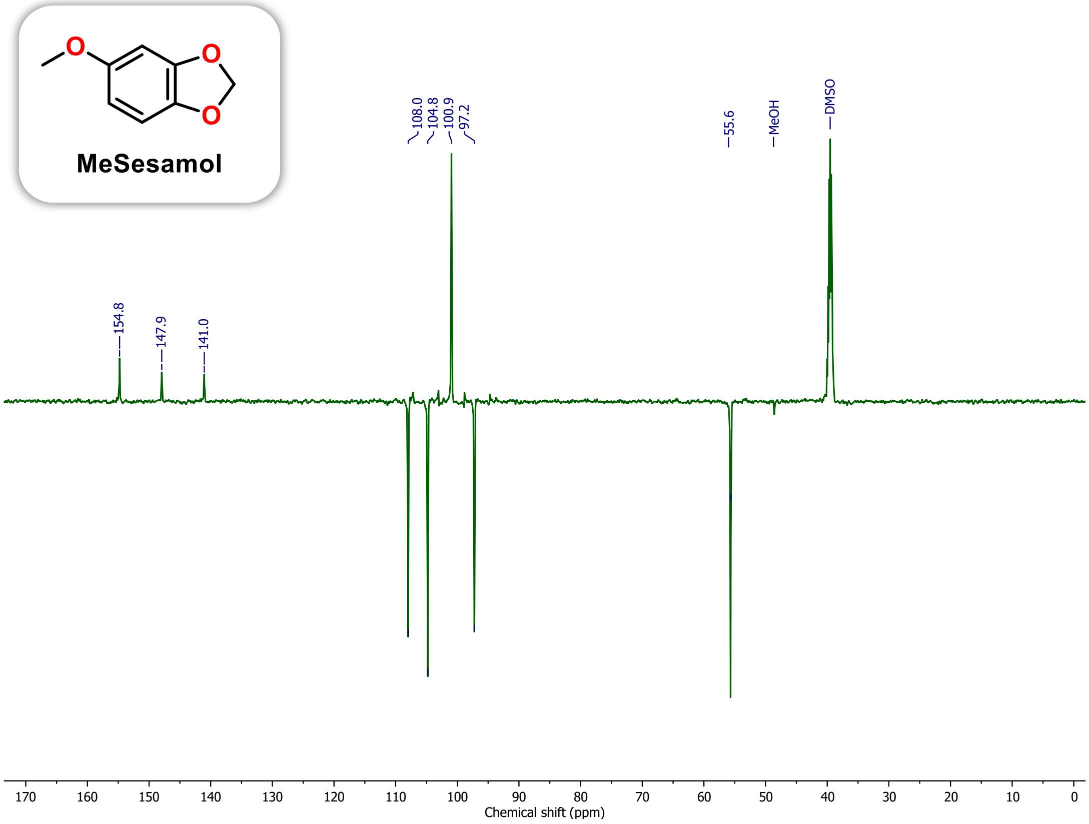

Figure S16 *DEPTQ spectrum of MeSesamol in DMSO-d_6*

7.4. Investigation of molecular interactions between MeSesamol and FDCA

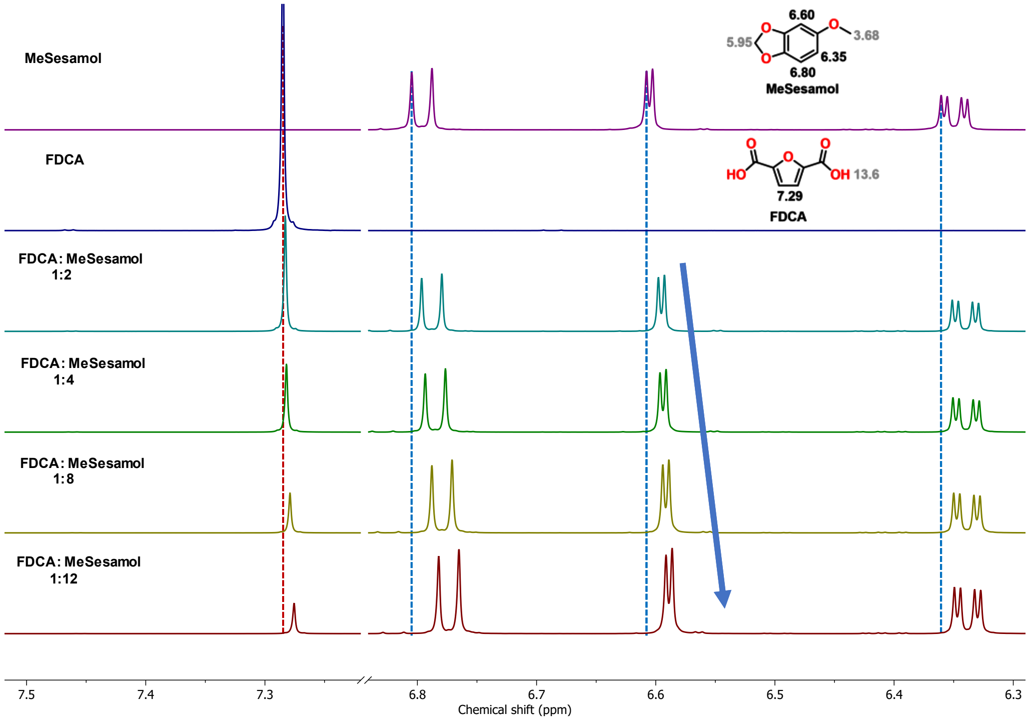

Figure S17 *Changes in the ^{1}H NMR chemical shift of FDCA in the presence of MeSesamol at different molar ratios in DMSO-d_6*

7.5. NMR spectra of PEF gained by polymerization

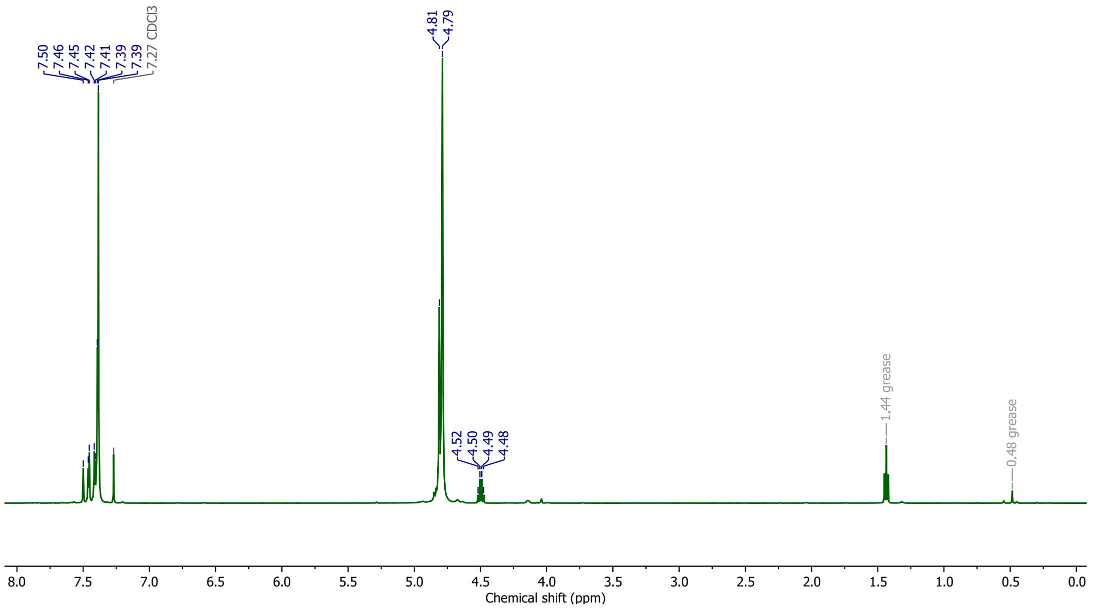

Figure S18 *^{1}H NMR spectrum of PEF gained by polymerization in TFA:CDCl$_3$ (1:1)*

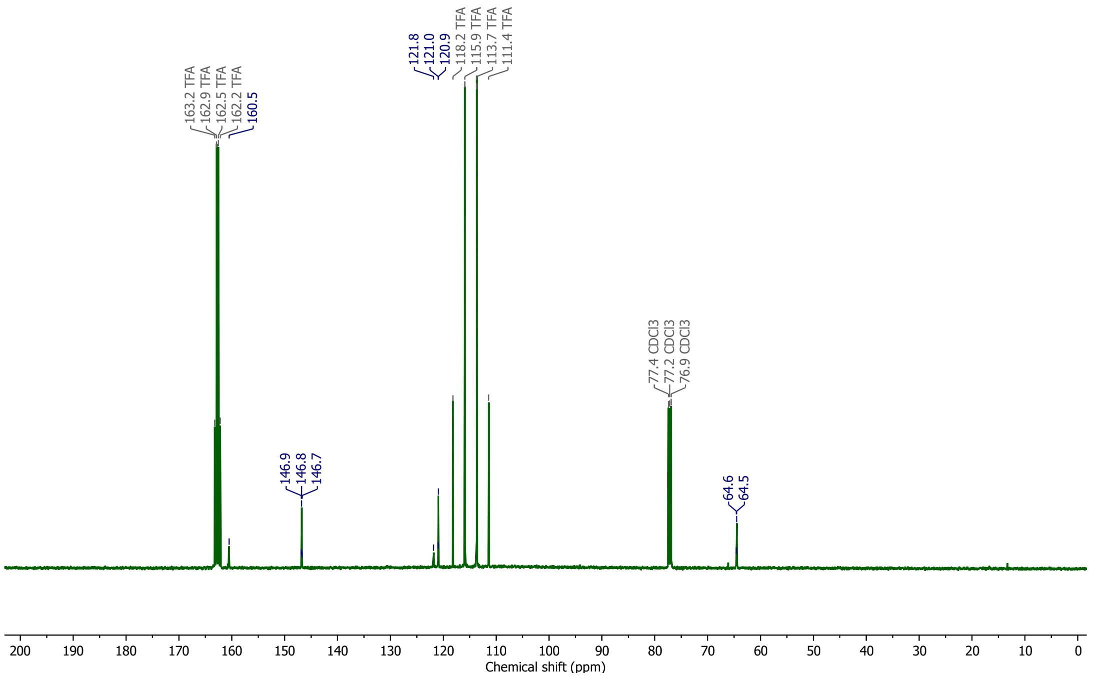

Figure S19 *^{13}C NMR spectrum of PEF gained by polymerization in TFA:CDCl$_3$ (1:1)*
